# Multi-modal comparison of molecular programs driving nurse cell death and clearance in *Drosophila melanogaster* oogenesis

**DOI:** 10.1371/journal.pgen.1011220

**Published:** 2025-01-03

**Authors:** Shruthi Bandyadka, Diane P. V. Lebo, Albert A. Mondragon, Sandy B. Serizier, Julian Kwan, Jeanne S. Peterson, Alexandra Y. Chasse, Victoria K. Jenkins, Anoush Calikyan, Anthony J. Ortega, Joshua D. Campbell, Andrew Emili, Kimberly McCall

**Affiliations:** 1 Graduate Program in Bioinformatics, Boston University, Boston Massachusetts, United States of America; 2 Department of Biology, Boston University, Boston Massachusetts, United States of America; 3 Molecular Biology, Cell Biology and Biochemistry Graduate Program, Boston University, Boston Massachusetts, United States of America; 4 Department of Biochemistry, Boston University Chobanian & Avedisian School of Medicine, Boston Massachusetts, United States of America; 5 Department of Medicine, Boston University Chobanian & Avedisian School of Medicine, Boston Massachusetts, United States of America; 6 Division of Oncological Sciences, Knight Cancer Institute, Oregon Health & Science University, Portland, Oregon, United States of America; Tulane University School of Medicine, UNITED STATES OF AMERICA

## Abstract

The death and clearance of nurse cells is a consequential milestone in *Drosophila melanogaster* oogenesis. In preparation for oviposition, the germline-derived nurse cells bequeath to the developing oocyte all their cytoplasmic contents and undergo programmed cell death. The death of the nurse cells is controlled non-autonomously and is precipitated by epithelial follicle cells of somatic origin acquiring a squamous morphology and acidifying the nurse cells externally. Alternatively, stressors such as starvation can induce the death of nurse cells earlier in mid-oogenesis, manifesting apoptosis signatures, followed by their engulfment by epithelial follicle cells. To identify and contrast the molecular pathways underlying these morphologically and genetically distinct cell death paradigms, both mediated by follicle cells, we compared their genome-wide transcriptional, translational, and secretion profiles before and after differentiating to acquire a phagocytic capability, as well as during well-fed and nutrient-deprived conditions. By coupling the GAL4-UAS system to Translating Ribosome Affinity Purification (TRAP-seq) and proximity labeling (HRP-KDEL) followed by Liquid Chromatography tandem mass-spectrometry, we performed high-throughput screens to identify pathways selectively activated or repressed by follicle cells to employ nurse cell-clearance routines. We also integrated two publicly available single-cell RNAseq atlases of the *Drosophila* ovary to define the transcriptomic profiles of follicle cells. In this report, we describe the genes and major pathways identified in the screens and the striking consequences to *Drosophila melanogaster* oogenesis caused by RNAi perturbation of prioritized candidates. To our knowledge, our study is the first of its kind to comprehensively characterize two distinct apoptotic and non-apoptotic cell death paradigms in the same multi-cellular system. Beyond molecular differences in cell death, our investigation may also provide insights into how key systemic trade-offs are made between survival and reproduction when faced with physiological stress.

## Author summary

Cell death occurs throughout development and homeostasis to removed damaged or obsolete cells. Typically dying cells are engulfed by neighboring phagocytic cells. In some cases, the phagocytic cells directly promote the death of neighboring cells, in a process called “phagoptosis.” The ovary of the vinegar fly, *Drosophila melanogaster*, provides a valuable model to investigate the differences between cell death mechanisms. The oocyte is normally supported by nurse cells during its development, and at the end of oocyte development the nurse cells undergo phagoptosis by surrounding follicle cells. Nurse cells can also undergo a form of cell death called apoptosis earlier in development in response to starvation, and apoptotic nurse cells are phagocytosed by follicle cells. To identify and contrast the molecular pathways underlying these morphologically and genetically distinct cell death paradigms, we compared their genome-wide transcriptional, translational, and secretion profiles. In this report, we describe the genes and major pathways identified in the screens and the striking consequences to *Drosophila melanogaster* oogenesis caused by genetic disruption of these genes. To our knowledge, this study is the first of its kind to comprehensively characterize two distinct apoptotic and non-apoptotic cell death paradigms in the same tissue.

## Introduction

Oogenesis is a tightly regulated interplay of germline and somatic cells conspiring to produce a viable egg. Like industrial production lines, organisms have evolved to reinforce egg production by adapting quality control measures, such as inspection checkpoints, corrective action, and elimination of defective egg chambers to optimize the health of eggs. Cell death is a requisite means to cull both sub-optimal products of oogenesis, as well as superfluous peripheral tissues that have completed their respective roles in supporting the oocyte on its way to maturation [1].

**Fig 1:**
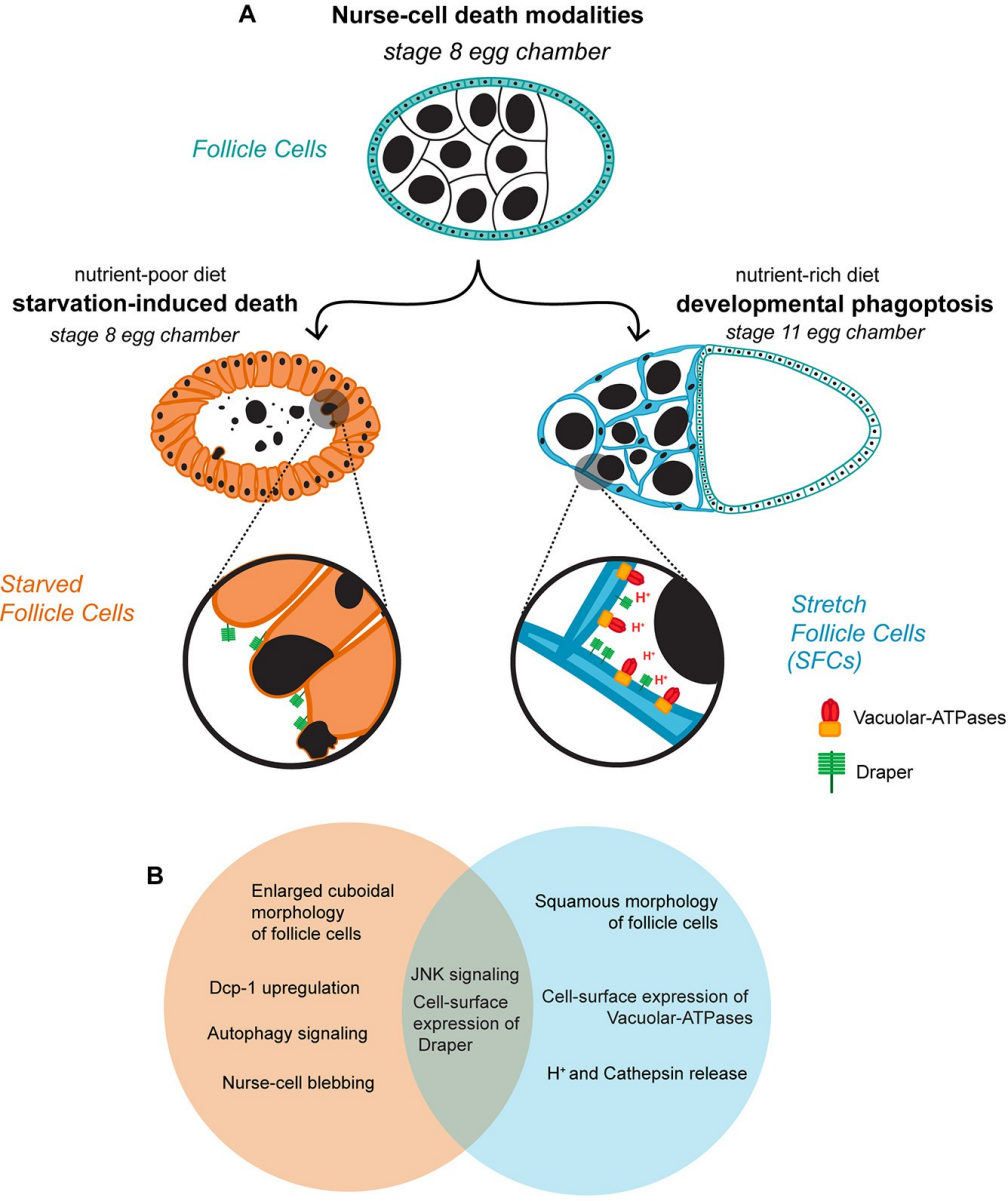
Nurse cell (NC) clearance is regulated apoptotically or non-apoptotically depending on environmental cues. (A) Schematic illustrating the different fates of egg chambers. A stage egg 8 chamber is completely surrounded by follicle cells (shown in teal). When the milieu is optimal and sufficient dietary protein is available, oogenesis proceeds (right side). A subset of the follicle cells differentiates into stretch follicle cells (SFCs, blue) and express the phagocytic receptor Draper and the proton pump Vacuolar-ATPase on their plasma membrane, ultimately acidifying the germline sister Nurse Cells (NCs, large black nuclei) externally to clear them. In contrast, when the organism is deprived of nutrition (left side), follicle cells enlarge in place (orange) and engulf the NCs, also by expressing Draper on their surfaces. In both death modalities, FCs clear NCs, albeit using different morphological configurations and biochemical pathways, providing a unique opportunity to study diverse cell death modalities in the same system (B) Venn diagram illustrating that developmental phagoptosis (blue) and starvation-induced death of NCs (orange) share key features such as the expression of Draper on the surface of the follicle cells and the activation of JNK signaling.

In *Drosophila melanogaster*, the egg chamber undergoes 14 well-defined stages of development, with significant physical and physiological interactions between the oocyte, the germline-derived Nurse Cells (NCs) and the somatic Follicle Cells (FCs) that form a monolayer encapsulating the germline [2,3] (Fig 1A). Multiple regulated cell death events mark the checkpoints during which both the egg’s competence and the suitability of the environment is surveyed. One such crucial checkpoint is encountered in mid-oogenesis during stages 7 through 9, when the decision to invest energy into yolk, chorion, and vitelline membrane deposition is made. As previously reported [4,5], seemingly innocuous physiological stressors such as short-term protein deprivation can induce a 60-fold difference in the rate of oogenesis. This diminished oviposition results in part from the degeneration of egg chambers in mid-oogenesis, which can be readily identified by the condensed and fragmented appearance of NC nuclei and the enlargement of FCs as they synchronously engulf the germline cells [6,7] (Fig 1A). Investigation into the underlying genetic factors has identified important roles for effector caspase *Dcp-1* [8–10] and autophagy [11–14], indicating that the germline is eliminated by a combination of apoptosis and autophagic mechanisms. Further, FCs express the phagocytic receptor Draper (Drpr) on their cell surfaces which promotes recognition and internalization of the dying germline, leading to the subsequent activation of the JNK signaling pathway to promote resolution of clearance [6,7,15].

When the milieu is optimal and any physiological insults are overcome, oogenesis proceeds through 14 stages. A marked difference in egg chamber morphology is observed as it enters the vitellogenic phase in mid-oogenesis after stage 8. A majority of the FCs encapsulate the oocyte, referred to as Main Body FCs (MBFCs), and a subset of them flatten and elongate to become Stretch Follicle Cells (SFCs) and extend into the spaces between the NCs [16] (Fig 1A). These later stages of oogenesis culminate in the nurse cells undergoing developmentally programmed non-apoptotic cell death. Stage 11 is defined by nurse cell “dumping”, in which maternal mRNAs, proteins and organelles from the NCs are deposited into the transcriptionally quiescent oocyte via ring canals, leaving behind the NC nuclei and a small amount of cytoplasm [3]. By stage 13, the NC nuclei begin to be eliminated non-autonomously and asynchronously by SFCs, in a process morphologically and physiologically distinct from starvation-induced NC clearance [17]. At the close of oogenesis at stage 14, all the NCs are cleared away, leaving only the fully developed oocyte.

Developmental NC death is controlled non-cell-autonomously and mediated by SFCs. Genetic ablation of SFCs revealed that SFCs were required for NC dumping, death, and clearance [18], while disruption of caspases and autophagy-related genes failed to prevent NC death, indicating that developmental NC death occurs through non-apoptotic and non-autophagic mechanisms [18,19]. However, the usual suspects in engulfment—*drpr*, *Ced-12*, and the JNK signaling pathway, were found to be required for NC death and clearance, indicating that the phagocytosis machinery is deployed by SFCs [18]. Subsequently, the NCs are externally acidified by SFCs targeting their lysosomal machinery, specifically Vacuolar-ATPases (V-ATPases), to their plasma membranes [20]. This form of cell death, where the phagocytosis apparatus of one cell is harnessed to kill another cell, which would otherwise continue to live, is known as phagoptosis [21,22]. Further details on the molecular mechanisms underlying both phagoptosis and starvation-induced NC death modalities have been reviewed more comprehensively elsewhere [1,7].

In both of these forms of nurse cell death and clearance, epithelial follicle cells are recruited for NC elimination, acting as non-professional, tissue-resident phagocytes, albeit in distinct morphological configurations (Fig 1A). While there are differences in genes required in the NCs for their death, the two death regimes share some components, specifically in the engulfment machinery acting in FCs, indicating that clearance requires the preferential activation or repression of specific sub-routines of death-associated signaling cascades (Fig 1B). The follicle cells could also be primed differentially by distinct external cues, arising both from NCs, in the form of “find me” and “eat me” signals [23], as well as from the extracellular environ, shaped in part, by the nutrient sensing pathway. How follicle cells in mid-oogenesis are able to make context-driven decisions to steer the fates of nurse cells, and of themselves, remains to be determined.

To determine how follicle cells are deployed differently during the two types of NC death, we took a multimodal approach by capturing the “translatome” and “secretome” of follicle cells in these diverse contexts. We performed Translational Ribosome Affinity Purification with RNA-sequencing (TRAP-seq), in addition to proximity labeling (HRP-KDEL) followed by Liquid Chromatography tandem mass-spectrometry (LC-MS/MS) of all follicle cells (AFCs) under fed and starved conditions, as well as the subset of SFCs, to identify the pathways that promote distinct nurse cell clearance processes. Further, we compared the translational profiles of fed AFCs and fed SFCs to their transcriptional profiles obtained by integrating two previously published single-cell RNAseq atlases of the fly ovaries [24,25] to identify genes that are exclusively under translational regulation. Our analyses and *in vivo* follow-up studies identified several key genes and pathways that play vital roles during the making of a healthy egg, such as maintaining the oocyte reserve, ensuring structural integrity of the egg chamber and regulating metabolic and signaling pathways. In summary, we have generated a multidimensional portrait of fly ovarian follicle cells with which we identify and distinguish key differences between follicle cell populations with a focus on regulators of NC death and clearance.

## Results

### Establishing the translatome, secretome, and transcriptome of AFCs and SFCs

**Fig 2:**
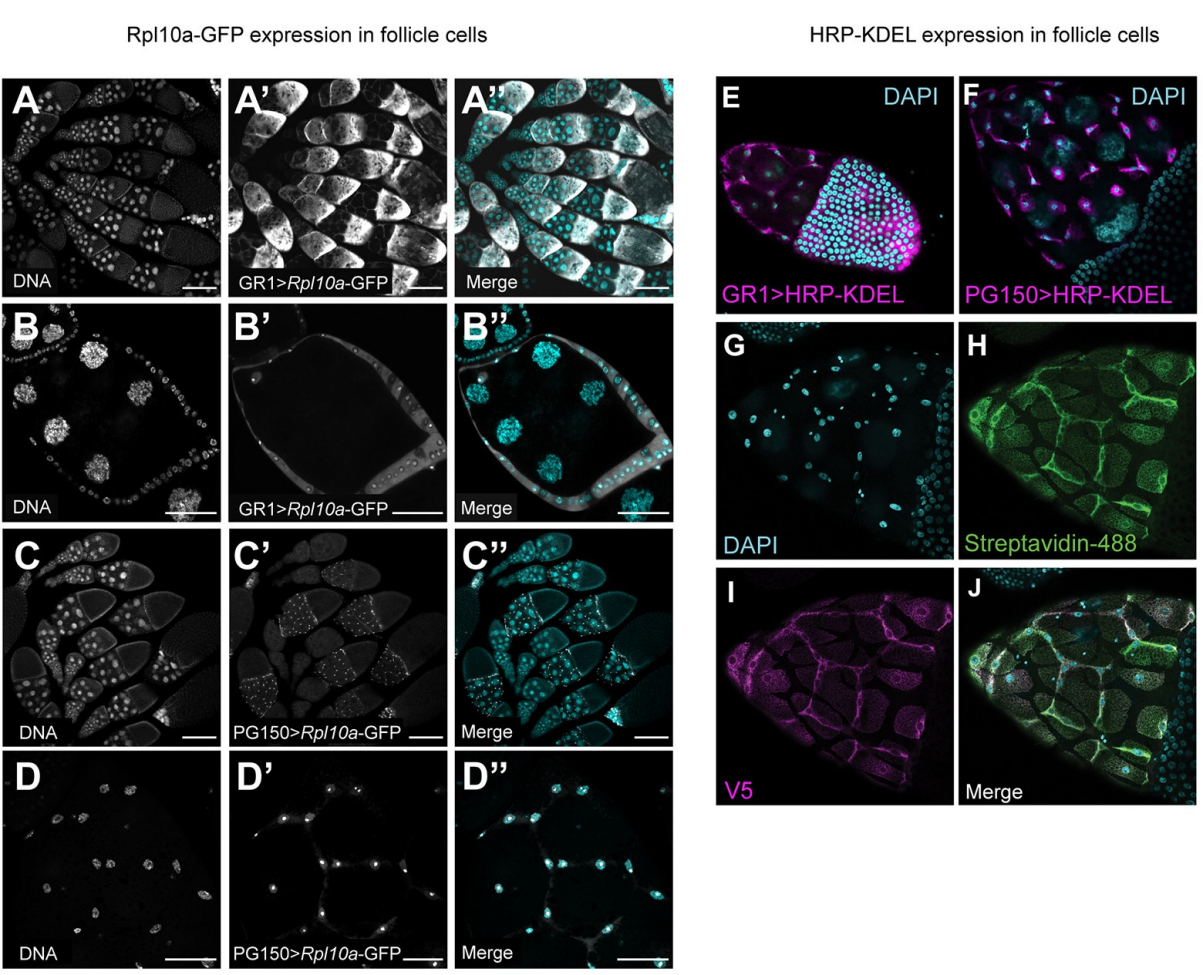
Expression of the RpL10Ab-GFP construct for TRAP-seq and the HRP-KDEL construct for LC-MS/MS. (A-D”) Egg chambers of indicated genotypes stained with DAPI. Merge shows DAPI in cyan and GFP in white. (A-A”) Several ovarioles expressing *GR1 > RpL10Ab-GFP* in AFCs. Younger egg chambers are located anteriorly (left). Scale bars = 200μ (B-B”) Expression of *GR1 > RpL10Ab-GFP* in AFCs of mid-stage egg chamber. Scale bars = 50μ (C-C”) Several ovarioles expressing *PG150 > RpL10Ab-GFP* in SFCs. Scale bars = 200μ (D-D”) Expression of *PG150 > RpL10Ab-GFP* in SFCs of stage 10 egg chamber. Scale bars = 50μ. (E-F) Egg chambers of indicated genotypes stained for DAPI (cyan) and anti-V5 (magenta) to detect HRP-KDEL. (E) *GR1>HRP-KDEL-V5* stage 9 egg chamber shows HRP-KDEL in all follicle cells. (F) *PG150>HRP-KDEL-V5* stage 10 egg chamber shows HRP-KDEL in stretch follicle cells only. (G-H) *PG150>HRP-KDEL-V5* stage 11 egg chamber with (G) DAPI, (H) biotinylated proteins (Streptavidin-488, green), and (I) HRP-KDEL (anti-V5, magenta). (J) Merge demonstrating that protein biotinylation pattern is restricted to cells expressing HRP-KDEL.

To capture the translatome or the set of all mRNAs undergoing translation in a cell, we performed TRAP-seq [26,27], by expressing GFP-tagged large ribosomal subunit 10a in follicle cells using the GAL4/UAS system [28,29]. We then performed immunoprecipitation to capture ribosome-bound mRNAs and subsequently sequenced and analyzed the transcripts. We used the *GR1-GAL4* strain to express RpL10Ab-GFP in all follicle cells after stage 3 of oogenesis (which we refer to as AFCs) (GR1>*RpL10Ab*-GFP) and the *PG150-GAL4* strain to express RpL10Ab-GFP in SFCs (PG150>*RpL10Ab*-GFP). RpL10Ab-GFP was confirmed to localize to the cytoplasm and nucleolus of the intended FC subtypes corresponding to the two GAL4 drivers (Fig 2A–2D”).

To establish the secretome or the set of all trans-membrane and secreted proteins of the follicle cells, we took a tissue and organelle-specific proteomics approach by combining the GAL4/UAS system with proximity-dependent protein labeling, followed by LC-MS/MS. Proteins fated to be secreted or trans-membrane are trafficked through the endoplasmic reticulum (ER) and Golgi network. To isolate and identify these proteins, we biochemically tagged and enriched proteins in the ER by expressing a genetically tagged fusion construct downstream of UAS, carrying horseradish peroxidase (HRP) fused to the ER-retention signal KDEL, along with secretion signal IgK leader sequence (ss) and the V5 epitope [30]. We expressed ss-HRP-KDEL-V5 in follicle cells, where it localizes to the ER. Like the translatome, we used GR1-GAL4 to express ss-HRP-KDEL-V5 in all follicle cells (GR1>ss-HRP-KDEL-V5) (Fig 2E) and PG150-GAL4 to express ss-HRP-KDEL-V5 in SFCs (PG150>ss-HRP-KDEL-V5) (Fig 2F). Next, we exposed dissected ovaries to biotin-phenol substrate and a brief pulse of H_2_O_2,_ which catalyzes the biotinylation of the proteins in the vicinity of HRP. We confirmed that the biotinylation is HRP-specific with α-V5 and streptavidin-488 staining (Fig 2G–2J) and additionally verified that the biotinylation is contingent on the availability of the biotin-phenol substrate and H_2_O_2,_ (S1A and S1B Fig). We then performed streptavidin enrichment followed by on-bead trypsin digestion and LC-MS/MS on the samples in two batches.

**Fig 3:**
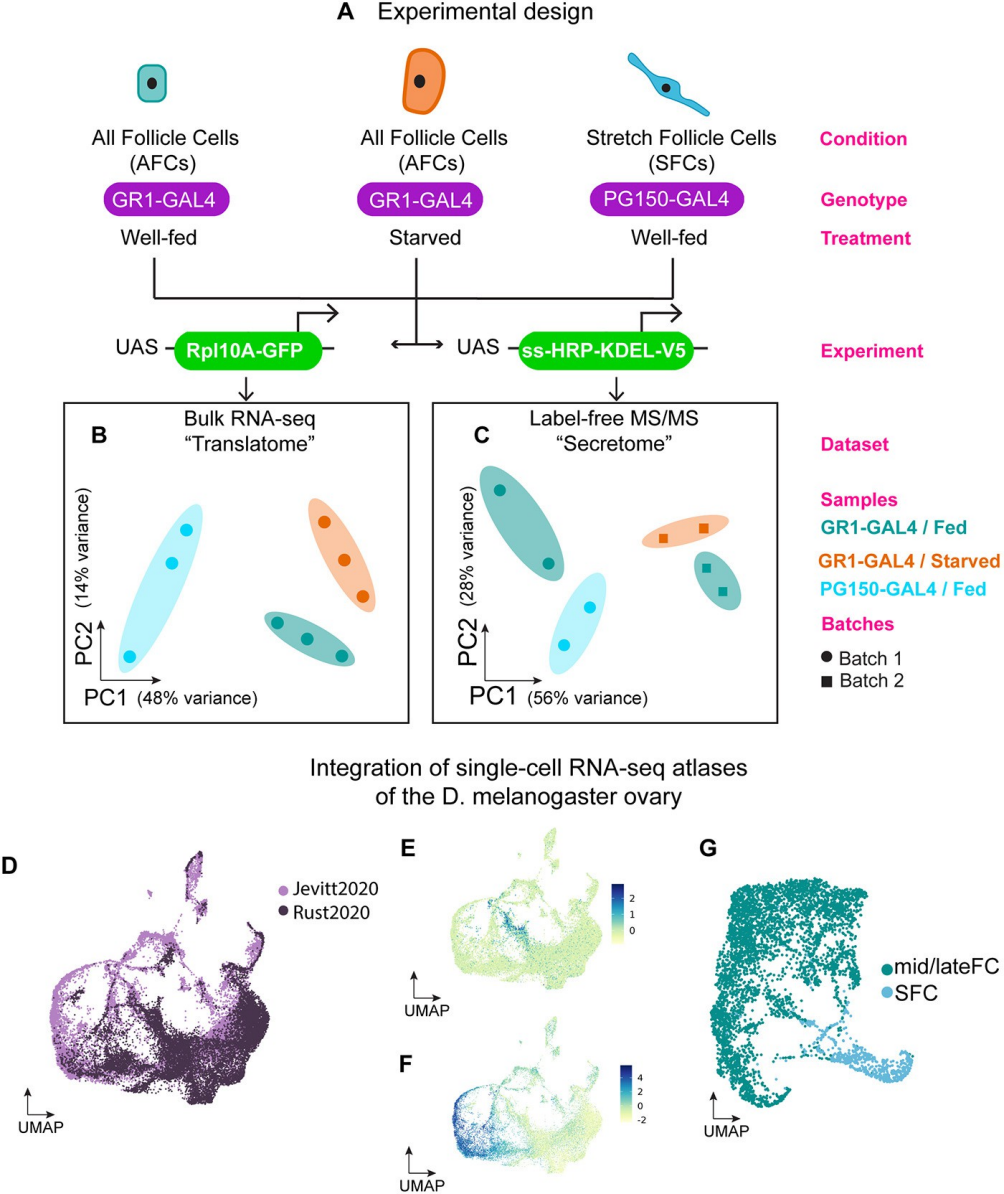
Establishing the translatome, secretome, and transcriptome of AFCs and SFCs. (A) Schematic illustrating the experimental design for identification of the translatome and secretome from 3 conditions/cell types. *GAL4* drivers *GR1* and *PG150* were used to drive expression of *RpL10Ab-GFP* in AFCs (fed or protein-starved) and SFCs. The resulting GFP-conjugated ribosomes were immunoprecipitated and the mRNAs bound to the ribosomes were sequenced to produce the translatome. Similarly, to generate the secretome, *GR1* and *PG150* were used to drive the expression of *ss-HRP-KDEL-V5* in AFCs and SFCs. HRP-KDEL expressed in cells localizes to the endoplasmic reticulum and biotinylated when incubated with biotin-phenol and H_2_O_2_. The tagged proteins were subsequently isolated using streptavidin beads and analyzed via LC-MS/MS to produce the secretome. (B) PCA Dimension reduction of aggregated gene-level transcript counts in translatome bioreplicates. (C) PCA of peptide intensity values of secretome bioreplicates. (D) UMAP embedding of Seurat RPCA integrated cells from publicly available single-cell RNA-seq atlases of the *Drosophila melanogaster* ovaries [24,25]. Cells are colored by the dataset of origin. (E) UMAP embedding of the integrated ovary datasets colored by SFC module score. To identify cells most likely to be stretch follicle cells in the integrated dataset, an aggregate score comprising the expression values of canonical SFC markers used in the published individual datasets were computed and projected on to the UMAP. (F) UMAP embedding of the integrated ovary datasets colored by mid/late follicle cell module score. To identify cells most likely to be mid/late follicle cells in the integrated dataset, an aggregate score comprising the expression values of canonical mid/late follicle cell markers used in the published individual datasets were computed and projected on to the UMAP. (G) Cells with a high SFC score (>1) and high mid/late follicle cell score (>2x) were extracted from the integrated dataset and subject to reclustering. Updated PCA and UMAP embeddings were computed for the new mid/late follicle cell and SFC subset and clusters containing mid/late follicle cells and SFCs were identified after normalizing read counts in the new subset.

To identify differentially expressed genes from the TRAP-seq experiment, we generated biological triplicates corresponding to experimental groups of fed AFCs, fed SFCs and starved AFCs (Fig 3A), with each replicate averaging over 49 million reads (Fig 3B). Analysis with Salmon and DEseq2 identified 1133 differentially translated genes (FDR Adjusted p<0.05) associated with phagoptotic clearance of nurse cells by comparing fed SFC samples to the fed AFC baseline (S2 Table). Similarly, comparison of starved AFC samples to fed AFCs identified 99 differentially translated genes (FDR Adjusted p<0.05) (S3 Table). The functional roles played by these candidates is described in detail in subsequent sections. To identify differentially abundant proteins from the LC-MS/MS experiment, we generated two replicates of fed AFC samples corresponding to two replicates each of fed SFCs and starved AFCs that were generated in separate batches (Fig 3C). Stringent database (MaxQuant) based spectral searching identified ~2200 unique proteins in batch 1 samples, and ~1800 in batch 2 samples (S1C and S1D Fig). The *GR1-GAL4>UAS-HRPKDEL* dataset was compared to a *GR1-GAL4* control dataset using COMPLEAT (Protein COMPlex Enrichment Analysis Tool) and nine of the top ten enriched complexes were known ER protein complexes [44], demonstrating correct subcellular localization of HRP to the ER. After removing decoys (reversed protein sequences used to gauge the false discovery rate) and potential contaminants (e.g. trypsin), we obtained a high confidence set of 40 differentially abundant proteins in the SFCs comparison (S2 Table) and 14 proteins in the starvation-induced comparison (FDR Adjusted p<0.05) (S3 Table).

Next, we leveraged two previously published single-cell RNAseq atlases of the fly ovary [24,25] to obtain complete transcriptomic profiles of AFCs and SFCs, against which we compared our findings from the translatome and the secretome, as well identified gene regulatory programs with finer resolution. The Jevitt et al. atlas [25] comprises cells from egg chambers across all developmental time points. However, the Rust et al. atlas [24] does not include vitellogenic egg chambers but delineates precursors to SFCs. Therefore, we integrated the two datasets using Seurat Reciprocal PCA and obtained a reclustered subset of cells that include putative AFCs and SFCs across the developmental continuum (Fig 3D).

To identify AFC and SFC populations comparable to the translatome and secretome, we obtained a cell identity module score using Seurat *AddModuleScore* based on the expression of canonical AFC and SFC markers, such as *Yp1* and *cv-2* respectively (A-E in S2 Fig) [31]. For the scope of this study, we bisected the reclustered subset broadly into AFCs and SFCs and identified differentially expressed genes between the two groups and confirmed their concordance with findings from the original publications (Fig 3E–3G). We used the direction and magnitude of log_2_ fold change (LFC) values from this comparison (SFCs / AFCs) to contrast findings from the translatome and the secretome.

### Distinct genes contribute to overlaps in gene ontology (GO) terms enriched in SFCs and starved AFCs compared to fed AFCs

**Fig 4:**
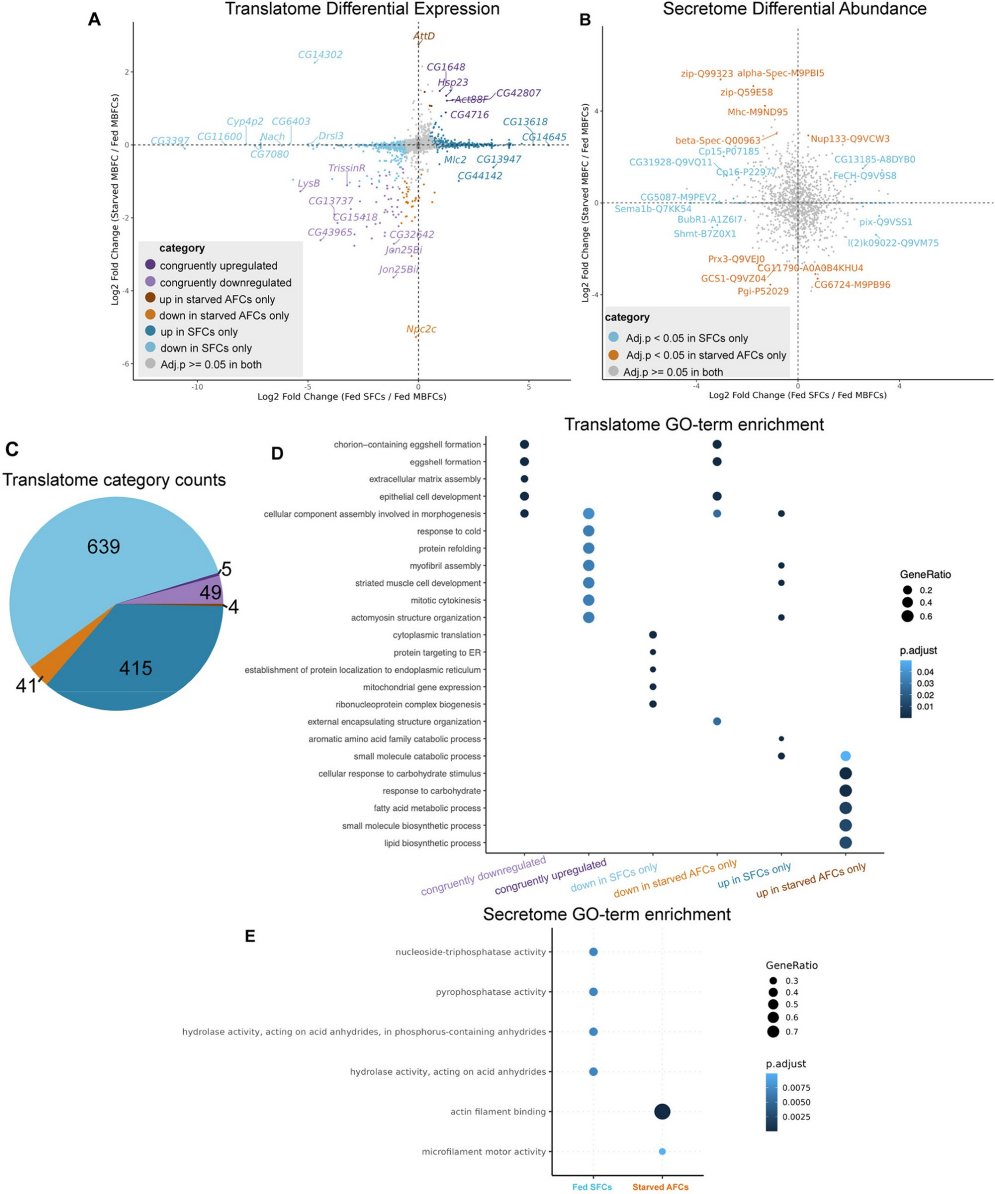
Functional enrichment of candidates identified in the translatome and secretome reveals pathways common and unique to phagoptosis (Fed SFCs/Fed AFCs) and starvation-death of NCs (Starved AFCs/Fed AFCs). (A) Scatterplot of differentially translated gene candidates in phagoptosis (Fed SFCs / Fed AFCs–x-axis) and starvation-death (Starved AFCs / Fed AFCs–y-axis) obtained using DESeq2. LFC differentials and FDR adjusted p-values from both analyses were compared to find genes congruently or uniquely regulated in both death modalities. Quadrants 1 and 3 indicate genes congruently up and down-regulated in phagoptosis and starvation-death respectively. Congruency was determined if the direction of LFC was the same and if the adjusted p-value was less than 0.05 in both. (B) Scatterplot of differentially abundant candidates in phagoptosis and starvation-death in the secretome. Proteins common to both death modalities were not found. UniProt identifiers along with gene symbols of detected proteins are specified. (C) Counts of genes regulated congruently and uniquely in phagoptosis and starvation-death of NCs in the translatome. Figure color legend same as in (A). (D) Dot plot of GO-terms enriched in the categories of differentially translated genes identified in (A and C). (E) Dot plot of GO-terms enriched in the secretome of fed SFCs and starved AFCs.

To identify shared and disjointed pathways between phagoptosis (fed SFCs vs. fed AFCs) and starvation-death of NCs (starved AFCs vs. fed AFCs), we compared the LFC estimates from both the translatome and the secretome (Fig 4A and 4B). For the sets of candidates identified in each dataset, we performed GO-term and KEGG pathway enrichment analysis [32]. We identified 5 congruently upregulated genes in the translatomes of SFCs and starved AFCs (Fig 4C) involved in cytoskeletal remodeling (GO:0030239, GO:0061640, GO:0031032) and 49 congruently downregulated genes, a majority of which are associated with vitelline membrane and chorion deposition (GO:0007304, GO:0030703) (Fig 4D). We also identified other genes associated with the same GO-terms but individual genes were unique to either SFCs or starved AFCs, highlighting the molecular differences underlying the similar processes that give rise to diverse phenotypic outcomes. The 415 genes uniquely upregulated in the SFC translatome were enriched for inositol and tyrosine metabolism (GO:0006020, GO:0006570), defense response (GO:0006952), and organic acid catabolism (GO:0016054). Accordant with our expectations, we found glucose homeostasis (GO:0042593), response to starvation (GO:0042594), and lipid metabolism (GO:0008610) over-represented exclusively in the starved AFC translatome. Notably, response to wounding (GO:0009611) was also over-represented in this group (S1 Table).

While the candidates identified in the secretome were different from those identified in the translatome in both SFCs and starved AFCs, we observed that the GO-terms over-represented in the corresponding comparisons were equivalent. For example, we found actin and microtubule associated proteins over-represented in the starved AFC secretome (GO:0003779, GO:0008017, GO:0005200). In the SFC secretome, we found enrichment of proteins involved in the hydrolysis of acid anhydrides (GO:0016817) (Fig 4E). We did not find any congruity in the differentially abundant proteins identified in SFCs and starved AFCs in the secretome.

We also compared the LFC values of differentially expressed genes between SFCs and AFCs identified from the translatome and the integrated scRNA-seq transcriptome. After filtering out genes with adjusted p value > = 0.05 in both datasets, we found 18 genes concordantly upregulated and 45 genes concordantly downregulated in SFCs (S2F–S2H Fig). A majority of these genes were involved in cytoskeletal remodeling and cell morphogenesis or in eggshell deposition. Overall, we found only *Vm32E* and chorion associated gene *CG12398* downregulated in SFCs across all 3 datasets (S2 Table).

### Reduced expression of eggshell deposition genes is accompanied by distinct changes to matrisome composition in SFCs and starved AFCs compared to fed AFCs

The *Drosophila* eggshell is a multi-layered extra-cellular matrix structure proximal to the oocyte, helping maintain egg chamber structural integrity. In mid and late oogenesis, AFCs and oocytes secrete sequentially, several proteins that comprise the vitelline membrane and the several layers that comprise the chorion [33–37]. In agreement with these findings, GO-term enrichment of our multi-modal dataset revealed that several genes involved in various eggshell deposition processes were downregulated in SFCs (compared to fed AFCs) at the transcriptome, translatome, and at the secretome level, confirming that the datasets are capturing the appropriate tissues, as well as confirming that eggshell deposition is a MBFC-specific trait. Further, we used the GLAD matrisome gene list [38,39] to annotate our multi-modal dataset and comprehensively identify patterns of regulation in specific structural and functional sub-classes of the matrisome. We found that vitelline membrane-associated genes *Vm26Aa*, *Vm26Ab*, *Vm26Ac*, *Vm32E*, *Vm34Ca*, and *Vml* were all congruently downregulated in fed SFCs compared to the fed AFC baseline in both the translatome and the integrated scRNA-seq transcriptomes. However, only *Vm32E* was significantly downregulated in SFCs in the secretome, while the directionality of fold change of other Vm genes in the secretome still remained concordant with the other datasets. Similarly, chorion-related genes *Cp15*, *Cp16*, *Cp19*, *Cp36*, *Cp18*, and *Cp38* were significantly downregulated in SFCs in the translatome, while only *Cp15* and *Cp16* were significantly downregulated in SFCs at the secretome level. Additionally, we observed other matrisome genes Semaphorin 1b (*Sema1b*) and BMP ligand *gbb* also downregulated by SFCs in the secretome, however this effect was not observed in the other datasets (S2 Table).

Previous studies have reported that starvation stress deters egg chambers from entering vitellogenesis [40], and stages 9–13 of oogenesis would be expected to be reduced in the starved samples. We observed diminished expression of *Vm32E*, *Vml*, and all chorion-related genes in protein-deprived AFCs, that we attribute to both reduced numbers of late-stage egg chambers in our starved samples and downregulation of vitelline membrane genes. In addition to these apical-matrix associated genes, we found other extracellular matrix (ECM)-affiliated genes such as ECM-regulator *PH4alphaPV* and immune-regulated glycoprotein *I(2)34Fc* to be downregulated in the translatome of starved AFCs (compared to fed AFCs). However, we did not observe a similar trend in their corresponding protein products in the secretome. These effects on the matrisome in both the SFCs and starved AFCs compared to fed AFCs provide strong evidence that our cell populations were correctly isolated in both the translatome and secretome.

### Ca^2+^-associated cytoskeleton regulators are required in SFCs for NC phagoptosis

We next aimed to identify genes with specific roles related to NC death and clearance in the two paradigms. During stages 9 and 10, the egg chamber further elongates and maintains an anterior-posterior axis while the oocyte grows tremendously to occupy over half the volume of the egg chamber. Keeping up with the increasing surface area of the egg chamber, anterior follicle cells elongate and flatten, acquiring the characteristic squamous morphology, cellular patterning, and identity of SFCs [41]. In contrast, during starvation-induced clearance, MBFCs undergo cytoskeletal rearrangements to enlarge in place and engulf NCs in a process that requires the GTPase Rac1 [6]. Rac1 is also required in SFCs even though acidification, but not engulfment of nurse cells has been observed in phagoptosis [18]. Several key questions related to cytoskeletal dynamics remain unanswered. Namely, how are follicle cells able to assume two different morphological configurations in phagoptosis and starvation-induced clearance of nurse cells? Are the same morphogenetic events required to surround and internalize nurse cell targets in both death paradigms?

**Fig 5:**
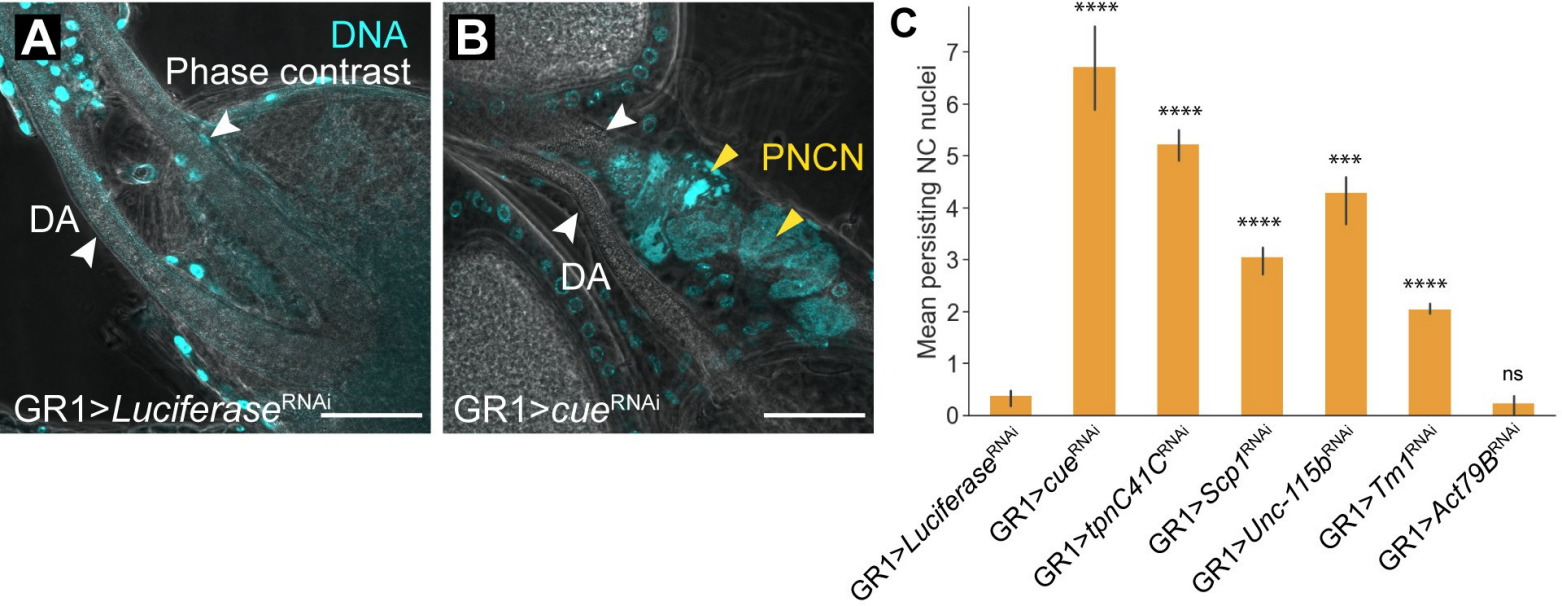
RNAi knockdown of Ca^2+^ associated cytoskeleton regulators leads to developmental NC clearance defects. (A-B) Representative images of anterior regions of stage 14 egg chambers from control (GR1 > *Luciferase* RNAi) and mutant (GR1 > *cue* RNAi) flies. DNA is labeled with DAPI (cyan). Stage 14 egg chambers are distinguished by two fully developed dorsal appendages (DA), indicated by white arrows. Persisting NC nuclei (PNCN) indicating a disruption to phagoptosis resulting from RNAi are marked by yellow arrows. Scale bars = 50μ. (C) Quantification of PNCN in stage 14 egg chambers. GR1 > *cue* RNAi demonstrates a strong PNCN phenotype, while the other knockdowns demonstrate a moderate to weak PNCN phenotype. At least 10 flies (20 ovaries) were used for each genotype. (*** p < = 5e-04, **** p < = 5e-05, ns p>0.05, one-sided independent t-test) Raw data available in S5 Table.

Towards this goal, we identified 26 cytoskeleton-associated genes upregulated in the translatomes of SFCs. In concordance with the SFC transcriptome, we found *Tm1* upregulated in the SFC translatome, along with several other calcium-dependent regulators of the cytoskeleton, such as *Tm2*, *up*, *Actn*, *Mlc1*, *Mlc2*, *bt*, *TpnC41C*, *Scp1*, *cue*, and *didum* (S2 Table). From the SFC secretome, we found a potential role for *armi* in late oogenesis, which closely associates with the microtubule cytoskeleton in egg chambers during early and mid-oogenesis, influencing egg chamber axial polarity during these stages [42]. Of note, we found actin encoding *Act88F* to be upregulated in both SFC and starved AFC translatomes. In addition, *Act79B* upregulation was exclusive to the SFC translatome. Because Ca^2+^ signaling plays a vital role in actin polymerization and phagocytic cup formation in other systems [43], we characterized the consequences of disrupting *TpnC41C*, *Scp1*, and *cue* by RNAi, using the GR1 driver. All 3 candidates demonstrated a persisting NC nuclei (PNCN) phenotype (Fig 5A–5C) similar to that observed in *draper* mutants [18], indicating their requirement in phagoptotic NC clearance. In addition, we performed RNAi of other cytoskeleton regulators and observed a weak PNCN phenotype (Fig 5C). These results suggest that phagoptosis, like phagocytosis, requires cytoskeletal rearrangements induced by Ca^2+^ signaling.

### Regulation of lysosomal machinery during phagoptosis of nurse cells

We previously reported that the lysosomal machinery, in particular V-ATPases, was required in SFCs for the acidification of nurse cells [20]. We also showed that RNAi of *Vha16-1* and *Vha100-2* in follicle cells leads to a strong persistence of NC nuclei in stage 14 egg chambers [20]. We therefore interrogated the integrated scRNA-seq atlas, translatome, and secretome datasets to systematically identify the isoforms of V-ATPase subunits expressed in SFCs and identify other lysosome associated genes that may assist V-ATPases with NC acidification. Our analysis of the integrated scRNA-seq dataset identified 15 genes associated with V-ATPases that were upregulated by SFCs at <1.5x LFC differential (S2 Table). By contrast, we found only *Vha44* to be upregulated by SFCs in the translatome in concordance with the transcriptome. Paradoxically, we found *Vha68-1* to be upregulated by SFCs in the translatome, while *Vha68-2*, another gene isoform that codes for the same V-ATPase subunit (V1 catalytic domain, subunit A) to be upregulated in SFCs in the transcriptome. At the secretome level, none of the V-ATPase protein products showed a significant differential even though peptides of various subunits were identified. We expressed RNAi against several V-ATPase genes using the *GR1-GAL4* driver, and identified a strong PNCN phenotype from *Vha100-1* RNAi and a moderate PNCN phenotype from *Vha44* RNAi [44] (Table 1). We also observed mid-stage death of egg chambers from RNAi of *Vha68-2* and *VhaAC39-1*, leading to a complete lack of mature eggs [44] (Table 1), indicating a requirement for these genes earlier in oogenesis that prevented analysis of their SFC function with this GAL4 driver. In addition to V-ATPases, we identified 18 lysosome-associated genes enriched in the SFC translatome. These include a transmembrane lysophospholipase *sws*, *g* (*garnet*) which encodes a subunit of lysosomal adapter protein complex AP-3, and *psidin*, which also affects lamellipodial dynamics in border cells, as well as initiates humoral immune response [45,46]. Likewise, the SFC secretome included 3 lysosomal proteins Plekhm1, FeCH, and CG43693. Notably, mutations in the human ortholog of Plekhm1 have been implicated in autosomal recessive osteopetrosis [47], which results from improper lysosomal acidification and resorption of the extracellular bone matrix [48], a mechanism reminiscent of SFC acidification of nurse cells. Loss of *Plekhm1* in follicle cells resulted in weak PNCN and excessive egg chamber degeneration in mid-oogenesis (Table 1). Our findings of differentially expressed lysosome-associated genes is consistent with our previous genetic analysis showing a strong requirement for lysosomal proteins in acidification of NCs during phagoptosis [20].

**Table 1:** Summary of results from the *in vivo* validation of candidates identified from the translatome, secretome, and the integrated scRNA-seq datasets.

Gene Symbol	Enriched in	Support (fold change directionality^)	Gene-Ontology Annotation	Stock Number (allele)	Phenotype summary
*Nach*	fed AFCs	translatome(-)	Secreted/ Transmem	27262 (JF02566)	Weak PNCN, Dumpless
*Nach*	fed AFCs	translatome(-)	Secreted/ Transmem	62894 (HMJ24134)	Moderate PNCN, Dumpless
*to*	fed SFCs	translatome(+)	Secreted/ Transmem	27646*	None
*CG33470* & *IMPPP*	fed SFCs	translatome(+)	Secreted/ Transmem	28540 (HM05026)	None
*Listericin*	fed SFCs	translatome(+)	Secreted/ Transmem	33058*	None
*Listericin*	fed SFCs	Translatome(+)	Secreted/ Transmem	33056*	None
*CG13998*	fed AFCs	scRNA-seq(-), translatome(-)	Secreted/ Transmem	34599 (HMS01073)	Moderate PNCN
*Drsl4*	fed SFCs	translatome(+)	Secreted/ Transmem	57186 (HMC04568)	Dumpless, Weak PNCN
*CG31659*	fed AFCs	translatome(-)	Secreted/ Transmem	57203 (HMC04586)	None
*CG13618*	fed SFCs	translatome(+)	Secreted/ Transmem	57749 (HMC04942)	Dumpless, Weak PNCN
*CG14645*	fed SFCs	translatome(+)	Secreted/ Transmem	60068 (HMC05061)	Weak PNCN
*CG31999*	fed SFCs	translatome(+)	Secreted/ Transmem	31587 (JF01162)	Weak PNCN, Dumpless
*LysB-Pdelta*	fed AFCs	translatome(-)	Secreted/ Transmem	[61]#	several Dumpless
*TotC*	fed AFCs	translatome(-)	Secreted/ Transmem	62159 (HMC05166)	None
*magu*	fed SFCs	scRNA-seq(+), translatome(+)	Secreted/ Transmem	51169 (HMJ21055)	Dumpless
*magu*	fed SFCs	scRNA-seq(+), translatome(+)	Secreted/ Transmem	41641 (GL01223)	Dumpless, Moderate PNCN
*alphaTry*	fed AFCs	translatome(-)	Secreted/ Transmem	51824 (HMC03396)	Dumpless
*thetaTry*	fed AFCs	translatome(-)	Secreted/ Transmem	53362 (HMC03591)	None
*Mur18B*	fed SFCs	translatome(+)	Secreted/ Transmem	56957 (HMC04397)	Dumpless, constricted egg chambers
*CG14259*	fed SFCs	translatome(+)	Secreted/ Transmem	57196 (HMC04578)	Weak PNCN, Dumpless
*CG31997*	fed SFCs	translatome(+)	Secreted/ Transmem	57517 (HMC04832)	Weak PNCN, Dumpless, Shriveled unhealthy stage 14 egg chambers
*wat*	fed SFCs	translatome(+)	Secreted/ Transmem	67801 (HMS05639)	Dumpless, abnormal NC nuclear morphology
*LBR*	fed SFCs	translatome(+), secretome(+)NS	Secreted/ Transmem	53269 (HMC02426)	severe Dumpless, midstage death
*Prip*	fed SFCs	translatome(+), secretome(+)NS	Secreted/ Transmem	50695 (HMC03097)	Moderate mid-stage death, Dumpless
*AttD*	starved AFCs	translatome(+)	Secreted/ Transmem	55979 (HMC04275)	None in fed, Higher mid-stage death in starved
*CG10163*	starved AFCs	translatome(+)NS	Secreted/ Transmem	32959 (HMS00754)	Higher mid-stage death in fed
*CG31926*	starved AFCs	translatome(+)	Secreted/ Transmem	64664 (HMC05699)	None in fed, none in starved
*Lsp1beta*	starved AFCs	translatome(+)	Secreted/ Transmem	27042 (JF02368)	None in fed, none in starved
*Drs*	fed SFCs	translatome(+)	Secreted/ Transmem	55391 (HMC04079)	Weak PNCN, Dumpless
*CG17192*	starved AFCs	translatome(+)	Secreted/ Transmem	56042 (HMC04350)	None in fed, none in starved
*CG11600*	fed AFCs	translatome(-)	Metabolic	66927 (HMS05393)	None
*CG3397*	fed AFCs	translatome(-)	Metabolic	44115 (HMS02837)	Weak PNCN, Dumpless
*Cyp6a17*	fed SFCs	translatome(+)	Metabolic	33887 (HMS00825)	None
*GstD10*	fed AFCs	translatome(-)	Metabolic	64678 (HMC05713)	Weak PNCN
*GstD7*	fed AFCs	translatome(-)	Metabolic	65961 (HMC06239)	Weak PNCN
*GstD1*	fed AFCs	translatome(-)	Metabolic	77338 (HMS05925)	Moderate PNCN, Dumpless
*GstD2*	fed AFCs	translatome(-)	Metabolic	77361 (HMS05960)	Weak PNCN, Dumpless
*GstD3*	fed AFCs	translatome(-)	Metabolic	48849 (GD17132)	Very Strong PNCN, dumpless
*CG12398*	fed AFCs	scRNA-seq(-), translatome(-), and secretome(-)	Metabolic	62837 (HMJ23931)	None
*Prosalpha3*	fed SFCs	scRNA-seq(+)& translatome(+), but enriched in Fed AFCs in secretome(-)	Metabolic	77145 (HMS05889)	Strong PNCN, stunted dorsal appendages, weak to moderate midstage death, severe arrested development, displaced and pyknotic NC nuclei, NCs in corpus luteum in fed. Severe midstage death and other previously listed phenotypes in starved
*CG6277*	starved AFCs	translatome(+)	Metabolic	33958 (HMS00915)	None in fed, none in starved
*CG6277*	starved AFCs	translatome(+)	Metabolic	51030 (HMJ21157)	None in fed, none in starved
*CG17633*	starved AFCs	translatome(+)	Metabolic	60043 (HMC05036)	None in fed, none in starved
*CG6508*	starved AFCs	translatome(+)	Metabolic	64953 (HMC05827)	None in fed, none in starved
*Jon25Bii*	starved AFCs	translatome(+)	Metabolic	55344 (HMC04031)	None in fed, none in starved
*Vps25*	fed AFCs	translatome(-)	Metabolic	26286 (JF02055)	Strong PNCN [44]
*Vps25*	fed AFCs	translatome(-)	Metabolic	54831 (HMJ21550)	None
*Not1*	fed SFCs	scRNA-seq(+)& translatome(+)	Metabolic	32836 (HMS00526)	Moderate mid-stage death, stunted dorsal appendages
*PGRP-LC*	fed SFCs	translatome(+)	NF-KB/Imd pathway	33383 (HMS00259)	Moderate PNCN, constricted or fused egg chambers, distended oocyte, premature dorsal appendage formation, dumpless
*SPE*	fed SFCs	translatome(+)	NF-KB/Toll pathway	33926 (HMS00873)	Severe mid-stage death in fed, severe morphological defects
*PGRP-SD*	fed SFCs	translatome(+)	NF-KB/Toll pathway	60904 (HMJ22903)	Moderate PNCN, dumpless
*numb*	fed SFCs	translatome(+)	notch pathway	35045 (HMS01459)	Excessive midstage dying, distended or fused egg chambers, dumpless, stunted dorsal appendages
*wgn*	fed SFCs	translatome(+)	TNF-a pathway	55275 (HMC03962)	None [62]
*Dad*	fed SFCs	scRNA-seq(+) & translatome(+)	TGF-b pathway	33759 (HMS01102)	Dumpless, midstage death, fused egg chambers and distended SFC membranes
*Act79B*	fed SFCs	translatome(+)	cytoskeletal	36857 (HMS00303)	None
*Unc-115b*	fed SFCs	translatome(+)	cytoskeletal	40839 (HMS02005)	Moderate PNCN
*cue*	fed SFCs	translatome(+)	cytoskeletal	36875 (GL01033)	Strong PNCN
*Scp1*	fed SFCs	translatome(+)	cytoskeletal	31957 (JF02573)	Moderate PNCN
*tpnC41C*	fed SFCs	scRNA-seq(+) & translatome(+)	cytoskeletal	80889 (GS04793)	Moderate PNCN
*Tm1*	fed SFCs	scRNA-seq (+) & translatome(+)	cytoskeletal	43542 (GL01518)	Weak PNCN, Dumpless
*Hsp23*	fed SFCs & starved AFCs	scRNA-seq (+) & translatome(+)	Chaperone and heat shock proteins	44029 (HMS02745)	None in fed, none in starved
*Hsp23*	fed SFCs & starved AFCs	scRNA-seq (+) & translatome(+)	Chaperone and heat shock proteins	82961 (HMS06055)	None in fed, none in starved
*Hsp23*	fed SFCs & starved AFCs	scRNA-seq (+) & translatome(+)	Chaperone and heat shock proteins	30541*	Moderate PNCN in fed, none in starved
*Npc2c*	starved AFCs	translatome(+)	transport/binding	61315 (HMJ23149)	None in fed, none in starved
*His2B*	starved AFCs	translatome(+)	DNA binding	41881 (HMS02295)	Higher mid-stage death in fed and starved
*CG6403*	fed AFCs	translatome(-)	Unknown	55983 (HMC04279)	None
*Plekhm1*	Fed SFCs	Secretome (+)	Lysosomal	42065 (GD11978)	Weak PNCN, Excessive midstage death
*BomS1*	Fed SFCs	translatome(+)	Secreted/ Toll pathway	42599 (HMS02431)	Weak PNCN
*Npc2a*	Fed AFCs	translatome(-)	Metabolic/ secreted	38237 (HMS01681)	Dumpless
*senju*	Fed SFCs	translatome(+)	Transporter/ Toll pathway	13399 (GD902)	None
*BomS3*	Fed SFCs	translatome(+)	Secreted/ Toll pathway	29214 (GD14501)	None
*Vha100-1*	Fed AFCs	translatome(-)(NS)	Lysosomal	26290 (JF02059)	Strong PNCN [44]
*Vha100-1*	Fed AFCs	translatome(-)(NS)	Lysosomal	57860 (HMJ21907)	Strong PNCN [44]
*Vha44*	Fed SFCs	translatome(+)	Lysosomal	33884 (HMS00821)	Moderate PNCN [44]
*VhaAC39-1*	Fed SFCs	translatome(+)(NS) scRNA-seq (+)	Lysosomal	35029 (HMS01442)	Midstage death [44]
*Vha68-2*	Fed SFCs	translatome(+)(NS) scRNA-seq (+)	Lysosomal	34582 (HMS01056)	Midstage death [44]

Stocks are RNAi lines unless otherwise indicated (* = OE, # = CRISPR/Cas9 deletion). ^Directionality of LFC values are indicated along with the dataset supporting the finding (+ = upregulation, - = downregulation) NS = Adjusted P-value < 0.05

### The role of innate immune signaling in NC death and clearance

The innate immune response in *Drosophila* relies on two highly conserved NF-κB pathways—Toll and Immune Deficiency (Imd). When the organism is immunologically challenged, a class of circulating secreted molecules called peptidoglycan recognition proteins (PGRPs) recognize and bind pathogen associated molecular patterns (PAMPs), such as Lys-type or DAG-type peptidoglycans or β-glucan which are expressed on the pathogen [49,50]. Depending on the infecting pathogen, Toll or Imd signaling is triggered preferentially.

**Fig 6:**
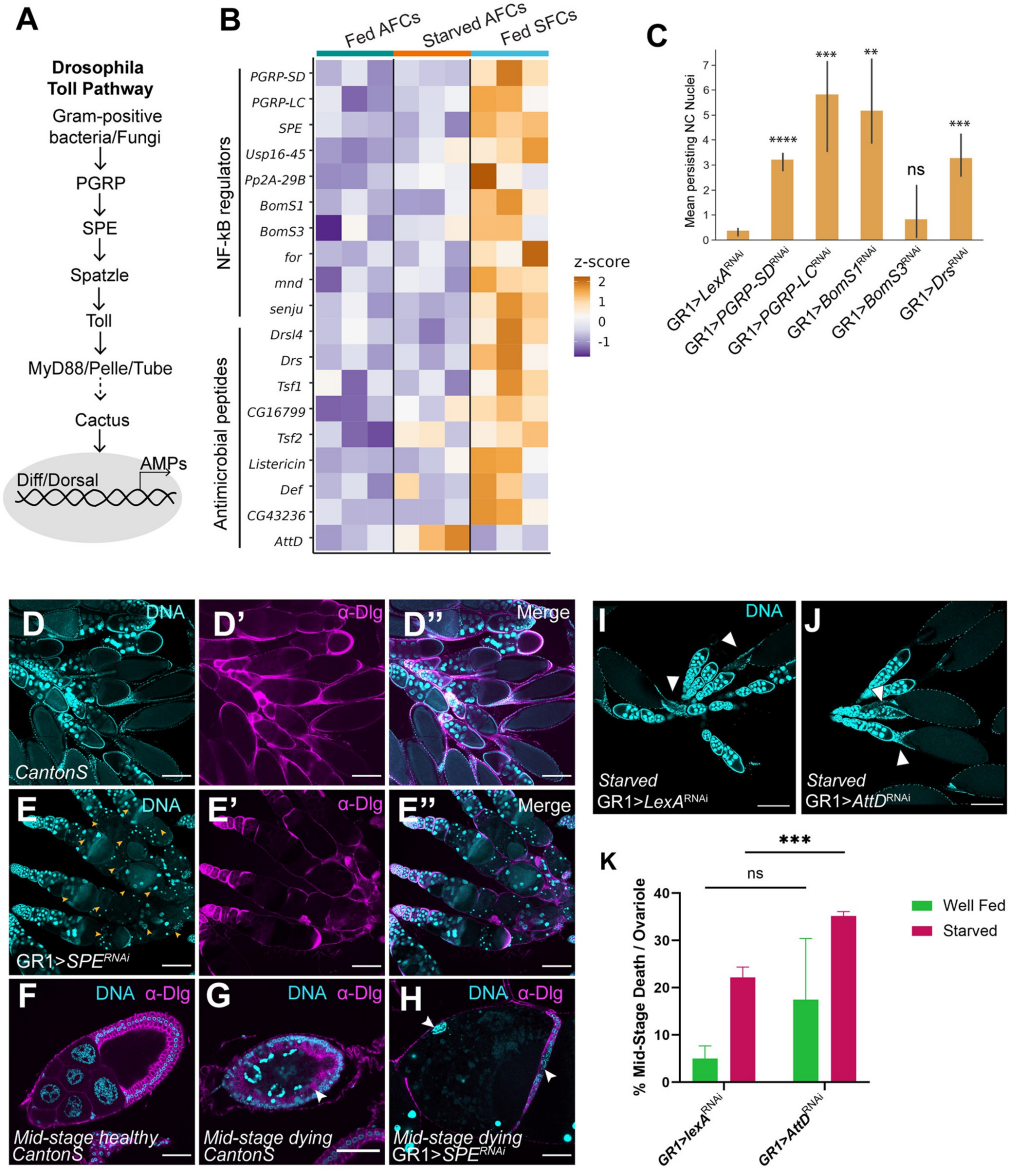
The role of innate immune signaling in NC clearance. (A) Schematic of *Drosophila* Toll pathway (B) Heatmap of scaled read counts of NF-κB regulators and antimicrobial peptide genes upregulated by fed SFCs or starved AFCs in the translatome. (C) Quantification of PNCN in stage 14 egg chambers from NF-κB or AMP knockdowns. (*** p< = 5e-04, **** p< = 5e-05, ns p>0.05, one-sided independent t-test). Significance levels are indicated for samples that have at least 3 replicates. (D) Egg chambers from wild-type fly ovaries stained with DAPI (cyan) and anti-Dlg (magenta) to label FC membranes. Scale bars = 200μ. (E) Egg chambers from *GR1>SPE RNAi* fly ovaries stained with DAPI and anti-Dlg. *GR1>SPE RNAi* ovarioles contain intact germaria and healthy early egg chambers but show wide-spread degeneration beginning around stage 6. Scale bars = 200μ. (F-H) Images of individual healthy and mid-stage dying egg chambers from wild-type ovaries and a mid-stage dying egg chamber from *GR1>SPE RNAi* stained with DAPI and anti-Dlg. The *GR1>SPE RNAi* degenerating egg chamber is lacking most of the follicle cell layer, indicated by white arrows. NC DNA in *GR1>SPE RNAi* is highly condensed and few are fragmented. Anterior-posterior polarity of the egg chamber is lost, with NCs extending into the posterior end, displacing the oocyte. Scale bars = 50μ. (I) *GR1>LexA RNAi* starved control shows sporadically degenerating egg chambers, Scale bars = 200μ. (J) *GR1>AttD RNAi* starved shows increased degeneration of egg chambers Scale bars = 200μ. (K) Quantitative analysis of midstage degenerating egg chambers with one-way ANOVA. (*** p < 0.0008, ns p> 0.1) Graph displays mean + SD with n > 24 females per genotype and condition. Raw data available in S5 Table.

Toll is typically activated upon infection by gram-positive bacteria or fungi, which initiates a serine protease cascade in host cells, whereby the Spatzle (Spz) precursor is activated by proteolysis by Spatzle Processing Enzyme (SPE), after which it binds to the membrane-bound Toll receptor [51,52] (Fig 6A). The intracellular Toll/Interleukin-1 receptor domain of the Toll receptor then binds to the adapter protein MyD88, which subsequently forms a complex with kinase Pelle and adapter protein Tube, leading to the phosphorylation and degradation of the *Drosophila* IκB factor Cactus [53,54]. This allows the translocation of the NF-κB family transcription factors Dif and Dorsal, ultimately activating the expression of genes encoding several antimicrobial peptides (AMPs) such as *Drosomycin* (*Drs*) and *AttacinA* (*AttA*) to combat infection [55]. When the cascade is triggered, the AMPs produced are embedded in the cell envelopes of pathogens, subsequently destabilizing and killing the pathogen [55]. Loss of *SPE* has been shown to impair *Drs* induction in response to microbial infection [52]. The Spz-Toll pathway also plays a role in development [56], and NF-κB pathways have been shown to be associated with neurodegeneration [50,57,58], so their roles extend beyond pathogen response.

Our analysis of the translatome revealed that SFCs showed elevated expression of not only the upstream peptidoglycan recognition molecules *PGRP-LC* (1x LFC*)* and *PGRP-SD* (1.5x LFC*)*, but also the protease *SPE* (1x LFC) and AMP *Drs* (2.8x LFC). Furthermore, we observed several other known and predicted NF-κB/Toll regulators and AMP genes upregulated in the SFC translatome (Fig 6B). To investigate the role of NF-κB/Toll signaling components in phagoptosis, we performed RNAi against *PGRP-SD*, *PGRP-LC*, *SPE*, *Drs*, and *Drsl4* and overexpressed *Listericin* (because an RNAi line was unavailable) using the GR1 driver. All of the transgenic lines, except *SPE* demonstrated a moderate or weak PNCN phenotype (Fig 6C). Surprisingly, we found that loss of *SPE* caused significant, widespread egg chamber degeneration in mid-oogenesis, resulting in near-complete loss of late-stage vitellogenic egg chambers. Almost all ovarioles in GR1>*SPE* RNAi mutants demonstrated an intact germarium and healthy early-stage egg chambers which began degenerating around stage 6 (Fig 6D and 6E). Antibody staining against Discs large (Dlg), a scaffolding protein that stains plasma membranes, showed that unlike starvation-induced degeneration of wild-type egg chambers in mid-oogenesis, GR1>*SPE* RNAi egg chambers neither demonstrated enlargement of MBFCs nor engulfment and clearance of NCs. MBFCs in GR1>*SPE* RNAi egg chambers instead were partially or completely missing in younger egg chambers, indicating that MBFCs are affected prior to NC degradation due to loss of *SPE*. Further, NC chromatin in GR1>*SPE* RNAi mutants appeared highly condensed, with some NC nuclei also undergoing fragmentation. Of note, the anterior-posterior polarity of egg chambers was also affected, with some dying NCs encroaching into the posterior end, displacing the oocyte. Several degenerating egg chambers did not show any detectable oocyte (Fig 6F–6H). While our experiments show that *SPE* is required for proper oogenesis, it is unknown whether the canonical Toll pathway is activated in follicle cells. While our bioinformatics analysis identified multiple components of the Toll pathway, including AMPs upregulated in the SFC translatome it remains to be determined if *SPE* acts in the canonical pathway to cleave Spz and if the AMPs upregulated have an immunogenic role in phagoptosis of NCs.

In contrast to *Drs* upregulation in SFCs, we found AMP gene *Attacin D (AttD)* to be upregulated in the starved AFC translatome. This was particularly intriguing as some Attacin genes encode glycine-rich AMPs, typically expressed in response to Gram-negative bacterial liposaccharides, regulated by the Imd pathway. Unlike the Toll pathway, which leads to the nuclear localization of Dif and Dorsal, the Imd pathway leads to the nuclear translocation of NF-κB transcription factor *Relish (Rel)*, which results from the phosphorylation of the N-terminus, as well as cleavage of C-terminus of Rel [50,59,60]. We wondered whether *AttD* functions as an AMP in starved AFCs to destabilize and promote apoptosis of NCs and if loss of *AttD* would attenuate egg chamber degeneration in response to starvation. Surprisingly, we found that RNAi knockdown of *AttD* in starved AFCs resulted in an increase in the number of mid-oogenesis deaths per ovariole. Additionally, our analyses showed that *AttD* RNAi in follicle cells did not cause significantly increased mid-stage death in well-fed flies, indicating that the transgenic construct is not sufficiently lethal to the egg chamber by itself (Fig 6I and 6K). This indicates an alternative role for *AttD* during starvation stress. One possibility is that AttD from AFCs of degenerating egg chambers functions as negative feedback, preventing or limiting apoptosis to preserve younger egg chambers. While *AttD* showed 2.8x LFC upregulation in the starved AFC translatome, we did not find upstream regulators of Imd or other Imd-associated AMPs to indicate the activation of the canonical Imd pathway (S3 Table). Interestingly *PGRP-LC*, which is typically associated with Imd activation was instead found upregulated in fed SFCs along with Toll regulators.

### *in vivo* RNAi screening of candidates involved in NC clearance

**Fig 7:**
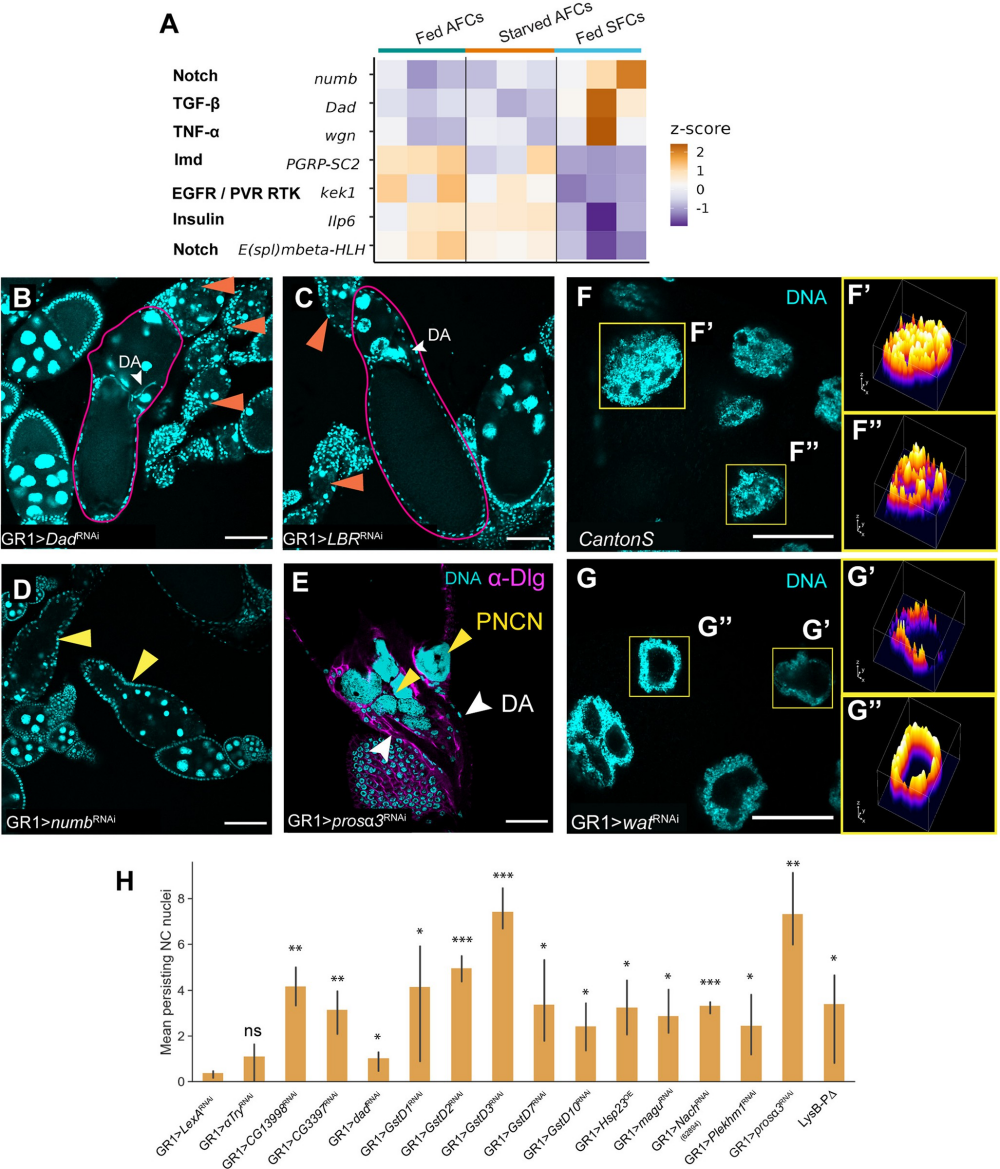
*in vivo* RNAi screening under fed condition. (A) Heatmap of scaled read counts of major signaling pathway genes that are significantly differentially regulated in SFCs. (B,C) *GR1>Dad RNAi* and *GR1>LBR RNAi* result in a “dumpless” phenotype (outlined in magenta), in addition to increased mid-stage death (orange arrows). Scale bar = 100μ. (D) *GR1>numb RNAi* results in mid-stage death accompanied by abnormal egg chamber morphology (yellow arrows). Scale bar = 100μ. (E) *GR1 > prosα3 RNAi* ovaries have a strong PNCN phenotype. Scale bar = 50μ. (F) NC nuclei in a stage 9 egg chamber from CantonS ovary. Scale bar = 50μ. (F’, F”) 3D surface plot of individual nuclei in stage 9 egg chamber from CantonS ovary after z-projection (max-intensity across stacks). Colors of peaks represent pixel intensity across z-stacks, with darker colors representing lower pixel intensity in the area (low DAPI staining) and lighter colors representing higher pixel intensity (robust DAPI staining). (G) NC nuclei in a stage 9 egg chamber from *GR1 > wat RNAi* ovary. Scale bar = 50μ. (G’, G”) 3D surface plot of individual nuclei in stage 9 egg chamber from *GR1 > wat RNAi* ovary after z-projection (max-intensity across stacks) reveals increased vacuolization. (H) Quantification of PNCN in stage 14 egg chambers of select candidates from Table 1. (* p < 5e-02, ** p < = 5e-03, *** p < = 5e-04, **** p < = 5e-05, ns p>0.05, one-sided independent t-test). Error bars indicate mean + S.D. across at least 3 replicates, with each replicate having at least 5 flies. Raw data available in S5 Table.

In addition to previously described gene families, we performed *in vivo* validation for a number of candidates identified across the 3 datasets presented herein (Table 1). A majority of the candidates we elected to follow up with were known secreted or transmembrane proteins, in order to identify any key components that may be involved in cell-cell communication in both death modalities. We also identified other candidates involved in major *Drosophila* signaling pathways to be differentially regulated exclusively in SFCs (Fig 7A). Specifically, we found *Dad* (*Daughters against dpp*) a component of the TGF-β/BMP signaling pathway to be enriched in SFCs in both the integrated scRNA-seq data and the translatome. Other genes in this pathway such as *cv-2* and *dpp* have been previously shown to be expressed in SFCs [16]. RNAi against *Dad* in follicle cells resulted in a weak PNCN phenotype, instead exhibiting a strong “dumpless” phenotype, in which NCs fail to dump their contents into the oocyte while the dorsal appendage continues to form (Fig 7B). We also observed several morphological defects, ranging from fused egg chambers and distended SFC membranes, to stunted dorsal appendages, indicating that *Dad* and the TGF-β/BMP pathway might also play a role in SFC differentiation and patterning in late-oogenesis. RNAi against several other candidates such as *Tm1*, *LBR* (Fig 7C), and *magu* also presented a dumpless phenotype (Table 1). RNAi against *numb* showed a range of phenotypes including dying and distended egg chambers (Table 1, Fig 7D). Of all the candidates we tested, GR1 > *prosα3 (proteasome α3 subunit)* and *GstD3* RNAi had the strongest persisting NC nuclei phenotypes (Fig 7E and 7H), with some stage 14 egg chambers retaining all 15 NC nuclei. In addition, *prosα3* RNAi also had egg chambers with several morphological defects, including stunted dorsal appendages, and increased mid-stage death even when fed a protein-rich diet. When starved, this genotype presented the same morphological defects and strong PNCN phenotype, in addition to increased midstage death. One of the most interesting mutant phenotypes we observed was in GR1>*wat* RNAi, which presented abnormal NC nuclei, starting around stage 8. NC nuclei in GR1>*wat* RNAi egg chambers had large areas devoid of DAPI staining, when compared to CantonS controls (Fig 7F and 7G). However, they did not appear to be pyknotic nor strongly persist in stage 14 egg chambers (Fig 7H). For all of the candidates tested, we qualitatively and quantitatively summarized mutant phenotypes, broadly categorized into 4 classes–NC clearance defects, NC dumping defects, egg chamber morphological and patterning defects, and excessive degeneration of egg chambers in mid-oogenesis (Fig 7H, Table 1).

Similarly, we investigated a number of genes differentially regulated in starved AFCs. We found that ovaries expressing RNAi against DNA binding gene *His2B*, heatshock protein *Hsp23*, and predicted triglyceride lipase *CG10163* had higher mid-stage death per ovariole than the starved matched control, while other knockdown lines had no significant detectable phenotype. (S3A–S3G Fig, Table 1). In addition to observing egg chamber health in starved mutants, we investigated whether the knockdown of the candidate genes was sufficient to induce egg chamber degeneration even in well-fed flies. We found that loss of *His2B* resulted in increased mid-oogenesis death per ovariole even in well-fed flies (S3G Fig).

## Discussion

The role of epithelial cells as non-professional phagocytes is well established. Previous studies have focused on singular models of apoptotic cell clearance by epithelial cells. To understand how the same cellular populations can participate in two distinct death paradigms, one apoptotic, and the other non-apoptotic, we characterized the multiple levels of gene regulation required to promote these events during *Drosophila* oogenesis. We hypothesized that instantaneous cellular responses in clearance, specifically during physiological insults such as nutrient-deprivation, would be orchestrated by translational bursts, rather than by energetically expensive transcriptional responses [63]. We therefore performed translational profiling of AFCs and SFCs using TRAP-seq and proteomic profiling using proximity labelling with mass spectrometry. Further, with the availability of single-cell gene expression profiles of AFCs and SFCs [24,25,41,64], we identified the sets of genes that may be post-transcriptionally controlled, by comparing candidates identified in scRNA-seq to those in TRAP-seq. Furthermore, whole-cell RNA sequencing data from phagocytic cells often contain contaminating mRNAs from the payload of engulfed cell corpses. By combining TRAP-seq and LC-MS/MS with the GAL4-UAS system, we afford finer control over both the cell types, as well as the mRNA species or peptides being profiled. By sequencing only those mRNAs that are bound to ribosomes, we could also disregard mRNAs that are primed for degradation and discover genes that are more likely to play a causal role in promoting clearance. Our analysis of the transcriptome, translatome, and secretome of follicle cells identified several commonalities and dissimilarities between SFCs and AFCs undergoing starvation, but did not identify a high degree of concordance between the datasets, as previously described by other studies [65]. These three datasets together highlight the different levels of gene regulation involved in promoting these distinct death modalities and serve as a resource for further in-depth investigation of NC death and clearance.

With this comprehensive resource, we first identified the distinct changes to the egg chamber matrisome during starved and well-fed conditions. This is accomplished by downregulating several components of the vitelline membrane, the chorion, and the yolk, which was captured in all three datasets. We also showed that glucose and lipid homeostasis is disrupted in the ovaries in response to nutrient deprivation, evidenced by the translational upregulation of *pepck1* and *FASN2* by starved AFCs. These results also reassured us that we were capturing the right cell populations. Several genes with functions in cytoskeletal remodeling were differentially expressed in both SFCs and starved AFCs, however only *Act88F* was common to them. A subset of the genes identified exclusively in the SFC translatome are known or predicted to encode proteins that bind calcium to promote myofibril assembly. *In vivo* validation of some of these candidates by RNAi revealed a strong PNCN phenotype, which supports the hypothesis that nonprofessional phagocytes, like professional phagocytes, utilize calcium signaling during cell corpse clearance [66]. We also showed for the first time that different AMPs are preferentially expressed during phagoptosis and starvation, *Drs* in the former and *AttD* in the latter. Moreover, SFCs upregulated the translation of multiple upstream regulators of the canonical Toll signaling pathway, including PGRP-LC, PGRP-SD, and SPE. However, it remains to be known if the Toll pathway has an immunogenic role in phagoptosis. Overall, we validated 76 transgenic lines *in vivo* (Table 1). Interestingly, several RNAi candidates with the GR1 driver affected NC survival rather than FCs. Because GR1 is not known to be active in NCs, it suggests that NC survival is determined by the health of the surrounding follicle cells.

The results presented herein also raise multiple questions. For example, we discovered that *AttD* translation is upregulated by starved AFCs and that loss of *AttD* exacerbates egg chamber degeneration. But what role does *AttD* play during starvation-stress and what factors are required upstream to activate *AttD*? Neither the translatome nor the secretome revealed a role for canonical members of the Imd cascade in the activation of *AttD* in starved AFCs. It would be worthwhile to test *in vivo* whether Imd pathway mutants, specifically the transcription factor *Relish*, affect *AttD* induction during starvation-stress. Because the *AttD* genetic and amino acid sequences are a phylogenetic outgroup among *Drosophila* Attacins, it is possible that *AttD* induction has a non-immunogenic role or even results from a heretofore unknown mechanism [67]. Hedengren et al. also noted that *AttD* lacks signal peptides and hypothesize that it could have a role in intracellular defense response to pathogenic challenge [59]. Moreover, there is evidence that suggests that the vast repertoire of *Drosophila* AMPs are used combinatorially, and that their specificity is determined by the source of the infection [68]. Taken together, we hypothesize that pathogenic and non-pathogenic insults may elicit diverse AMP responses. Characterizing the substrates required for preferential AMP induction in oogenesis would have implications beyond understanding how discriminatory immune responses are regulated. Nutrient availability is a key evolutionary selective pressure that influences life-history trade-offs between survival and reproduction at the individual level. Resource allocation constraints affect not only maternal health and fecundity, but also determine the offspring size and fitness. Ultimately, the molecular differences and similarities between the two death paradigms in oogenesis highlighted in this report take us a step closer to understanding and appreciating how cell death shapes and sustains life.

While this study is a valuable resource to generate several new hypotheses, our findings are constrained by the limited availability of samples. SFCs make up a small fraction of the egg chamber cellular demographic. In addition to low abundance of cells, capturing specific subsets of mRNAs and proteins might underrepresent species that are lowly abundant or those that occupy spatiotemporal niches, as well as those that undergo alternative modes of post-transcriptional regulation. For example, Drpr, which has been established to be essential for engulfment and has been shown to increase in engulfing FCs by immunostaining [6,18], was not reported to be upregulated by SFCs or starved AFCs in any of the datasets. Therefore, exploration of other means of regulation that explain the mechanism by which these notable genes are involved is warranted. Furthermore, our *in vivo* validation of the findings is constrained by the limited availability and potential imprecise targeting effects of RNAi constructs. While we expected our *in vivo* RNAi candidates to yield severe clearance defects leading to strong persistence of NCs, we only observed weak to moderate clearance defects in most candidates, in addition to a plethora of dumping and morphological defects (Table1). This could also be attributed to epistatic effects or could further indicate the presence of redundancy in clearance pathways. It is also likely that some of the candidates discovered have functions in follicle cells that are not associated with NC clearance. Additional confounding may result from the non-specificity of GR1 and PG150, as they are weakly expressed in the gut and Malpighian tubules respectively and a known issue with the GAL4 system. Finally, all *in vivo* RNAi experiments reported herein of candidates differentially regulated in SFCs were performed using the *GR1-GAL4* driver as it is expressed prior to SFC differentiation and shows strong effects driving genes that affect NC clearance [18]. Crosses performed with *PG150-GAL4* for RNAi of *SPE* (33926; HMS00873) and *Prosalpha3* (77145; HMS05889) did not yield any viable F1s even when raised at 18°C, despite producing strong phenotypes with *GR1-GAL4*. Further experiments beyond RNAi and overexpression are required to disentangle the specific contributions of these genes and their complex interactions to promote NC death and clearance.

## Materials and methods

### Fly husbandry

All flies were obtained from Bloomington (BDSC) or Vienna Stock Center, or other laboratories in the *Drosophila* community (Table 1). *GR1-GAL4* (gift from Dr. Trudi Schüpbach) was utilized to drive expression in all follicle cells and *PG150-GAL4* (gift from Dr. Ellen LeMosy) was used to drive expression in stretch follicle cells [69,70]. *GR1* is expressed in all FCs beginning in stage 3 egg chambers [6] including SFCs. *PG150* is expressed in the SFCs and centripetal cells. *UAS-Luciferase RNAi* (Bloomington stock #31603) or *UAS-LexA RNAi* (Bloomington stock #67946) were used as controls for all RNAi experiments [71] and the *UAS-RpL10Ab-GFP* line was BDSC #42681. All flies were raised on standard molasses/yeast/agar/cornmeal food. Long term fly stocks were stored at 18°C. Active stocks were raised at 25°C. Crosses were performed at 25°C. For the well-fed experimental condition, flies were aged 3 days at 25°C on standard cornmeal, molasses, and agar mixture, and then fed fresh yeast paste every 24 hours for 2 consecutive days before abdomens were dissected. For the protein starvation condition, flies were transferred for 16–20 hours to apple juice agar lacking yeast.

### Translatome library preparation and bulk RNA-seq

#### Fly abdomen collection

To collect enough ovaries for RNA sequencing and prevent RNA degradation, whole fly thorax and abdomens were collected from samples containing approximately 100 flies. Flies of the appropriate genotype were transferred to a 15 mL conical tube and submerged in liquid N2 for 1 min. The conical tube was then vortexed vigorously for 20 s to break flies apart and tissues were collected over a set of sifters to obtain thoraxes and abdomens. Once samples were processed, they were transferred to a 1.5 mL microcentrifuge tube and stored in a -80°C freezer for up to one month.

### Immunoprecipitation of ribosome-bound mRNAs

Ribosome immunoprecipitation was performed as per [72]. Fresh magnetic beads were prepared from the Dynabeads antibody coupling kit (Life Technologies) and conjugated to the19C8 anti-EGFP antibody (Memorial Sloan Kettering). Abdomens of 100 flies were homogenized on ice in 400 μl of homogenization buffer (9.1 mL nuclease free H2O (Ambion), 200 μL 1M HEPES-KOH pH 7.4 (USB), 750 μL 2M KCl (Ambion), 50 μL 1M MgCl2 (Ambion), 1 Complete Mini EDTA-Free Protease Inhibitor Cocktail Tablet (Roche), 5 μL 1M DTT (Sigma), 10 μL Super RNasin (Ambion), and 10 μL of freshly prepared 1000 mg/mL cycloheximide (Sigma)). Homogenized fly samples were then centrifuged for 30 min at 20,000g at 4°C. The supernatant was transferred to new pre-chilled 1.5 mL tubes. A 1:1 mixture of 300 mM 1,2-diheptanoyl-sn-glycero-3- phosphocholine (DHPC, Avanti) and 10% Igepal (USB) was added to the to the supernatant in a ratio of 1 part mixture: 4 parts supernatant. The solutions were mixed by inversion, incubated on ice for 5 min, then centrifuged for 5 min at >20,000g at 4°C. Cleared lysate was added to an aliquot of the bead suspension and incubated for 1 hr at 4°C with end over end rotation. The mixture was briefly centrifuged then magnetized for 2 min. After removing the buffer, beads were rinsed 6 times in 1 mL 0.35M KCl Wash Buffer (7.1 mL nuclease-free H2O (Ambion), 200 μL 1M HEPES-KOH pH 7.4 (USB), 1.75 mL 2M KCl (Ambion), 50 μL 1M MgCl2 (Ambion), 1 mL 10% Igepal (USB), 5 μL 1M DTT (Sigma), and 50 μL of freshly prepared 1000 mg/mL cycloheximide (Sigma)). After final rinse, beads were resuspended in 200 μl of nuclease-free water (Ambion) and RNA was extracted with TRIzol (Invitrogen). RNA quality was assessed with an Agilent RNA 6000 Bioanalyzer.

### cDNA library production and sequencing

The Illumina TruSeq RNA Sample Preparation v2 was used to convert 0.1–4 μg of precipitated mRNA to cDNA in preparation for RNAseq. Poly-T oligo-attached magnetic beads were used to isolate mRNA and the mRNA was fragmented and primed with random hexamers for cDNA synthesis using SuperScript II. Second strand cDNA synthesis was performed and in preparation for attaching the Illumina adaptor sequences, a single A nucleotide was added to the 3’ ends of the blunt fragments. Samples were run on an Agilent BioAnalyzer (BU Microarray core). Once the samples were validated by the core, they normalized, pooled, and sequenced on an Illumina NextSeq500. The sequencer was instructed to run 400 million 75bp single end reads.

### TRAP-seq QC, differential expression analysis, and functional enrichment

RNA-seq data from biological triplicates corresponding to well-fed and starved AFCs (*GR1-GAL4>UAS-RpL10Ab-GFP*), and well-fed SFCs (*PG150-GAL4>UAS-RpL10Ab-GFP*) were assessed for sequence quality using FastQC v0.11.7. Illumina sequencing adapters were computationally removed by the Boston University microarray sequencing core facility, where the sequencing was performed. Samples were evaluated for per-base Phred score and for overrepresented sequences. Reads that passed QC evaluation were aligned to the *Drosophila melanogaster* reference genome BDGP6.32 release 104 using Salmon v1.1.0 in the quasi-mapping mode. This step was parameterized to automatically infer sequence library type using the -l A flag, correct for fragment-level gc-bias, as well as perform selective alignment using–validatemappings.

The aligned reads in the salmon quant file format were then imported into DESeq2 to identify differentially translated genes. Genotype (AFC vs. SFC) and treatment (well-fed vs. starved) were both used as factors in the DESeq2 linear model design [73]. Log fold change estimates were obtained after applying the ApeGLM method for effect size shrinkage [74]. ClusterProfiler v4.2.2 and PANGEA were used to identify the pathways and gene ontology terms enriched in both comparisons [75,76]. GLAD webtool was used to obtain additional functional annotations [77].

### Secretome Sample Preparation and Liquid Chromatography-Tandem Mass Spectrometry

#### Molecular cloning of *UAS-ss-HRP-KDEL-V5*

pDisplay-ss-V5-HRPKDEL was obtained from Dr. Alice Ting [30]. The insert was PCR-amplified and cloned into the pENTR/D-TOPO cloning kit (Invitrogen) and pTW vector (*Drosophila* Genome Resource Center, RRID:DGRC_1129)) using Gateway cloning (Invitrogen). The plasmid was purified by Qiagen Midiprep kit, confirmed by sequencing and sent to BestGene (Chino Hills, CA) for injection into *Drosophila* embryos.

### Protein biotinylation using HRP-KDEL

Freshly dissected ovaries were incubated in 300 μL of 500 μM biotin phenol for 30 minutes at room temperature rotating. Samples were then rinsed with 1X PBS twice and the biotinylation reaction was initiated by adding 1 mM H_2_O_2_ in PBS to the samples for 1 minute and rotating at room temperature. Ovaries were quickly washed with quencher solution (10 mM sodium ascorbate, 5 mM Trolox (Sigma-Aldrich), 10 mM sodium azide, then lysed in 100 μL RIPA buffer with quencher solution for 5 min on ice. RIPA buffer was composed of: 50 μL 1M Tris-HCl, 150 μL 5M NaCl, 50 μL of 10% SDS, 250 μL of 10% Sodium Deoxycholate, 500 μL of 10% TritonX-100, 50 μL of 100X Protease Inhibitor (Sigma-Aldrich–P8849), 50 μL of 100 mM PMSF, 3.550 mL of diH20. Tissue was homogenized by motorized pestle and centrifuged at 16.1g for 10 min at 4°C. Clarified sample (clear middle layer) was transferred to a new tube and snap frozen in liquid nitrogen. This protocol was adapted from [78,79].

### Biotinylated protein pull-down

Frozen protein samples were thawed on ice. Meanwhile, 50 μL streptavidin magnetic beads (Pierce 88817) were washed with 1 mL of RIPA lysis buffer twice. The beads were subsequently incubated with 90 μL of protein lysate (about 550 μg of protein) and an additional 500 μL of RIPA buffer was added to facilitate rotation for 1 hour at room temperature. Beads were pelleted on a magnetic rack and the supernatant (flow-through) was collected on ice. After each wash, the magnetic beads were transferred into new tubes and washed twice with 1 mL RIPA buffer, once with 1 mL KCl, once with 1 mL 0.1 M Na_2_CO_3_, once with 1 mL 2 M Urea in 10 mM Tris-HCl (pH 8), and twice with 1 mL RIPA buffer. For the proteomic analysis, RIPA buffer was removed, and the beads snap frozen in liquid nitrogen and stored at -80°C. Otherwise, protein was eluted by boiling each sample in 30 μL of 3X protein loading buffer (containing DTT, Pierce) supplemented with 2 mM biotin (Sigma-Aldrich) for 10 min. After boiling, the beads were pelleted with the magnetic rack and the eluate collected for analysis by Western blot. Biotinylated proteins were probed on western blot with streptavidin-HRP (Thermo Fisher Scientific).

### Western blotting

Protein was isolated by lysing whole ovaries in 100 μL fresh ice-cold RIPA buffer for 5 minutes. After the 5-minute incubation, tissue was disrupted by using a motorized pestle until clumps were broken down (about 30 seconds). Samples were centrifuged for 10 minutes at 4°C at 16.1 relative centrifugal force (RCF). The clarified sample (clear middle layer only) was transferred to a new tube. Protein that was to be analyzed later was snap frozen in liquid nitrogen and stored at -80°C. Laemmli loading buffer with 2-mercaptoethanol (BME, Bio-Rad) was added to the protein samples (1:3), boiled at 95°C and then immediately stored on ice. Samples were run on a 10% resolving gel, transferred to nitrocellulose and protein was detected by enhanced chemiluminescence (ECL, Thermo Fisher Scientific).

### On-bead trypsin digestion and LC-MS/MS

Beads from biotinylated protein pull-down were washed with 100 mM triethylammonium bicarbonate. Peptides were eluted from beads by on-bead trypsin digestion with 1μg Trypsin (Pierce) in 100 mM triethylammonium bicarbonate overnight rotating at 37°C. Peptides were desalted using C18 ZipTip (Millipore) and subjected liquid chromatography coupled to tandem mass spectrometry on a Q Exactive HF-X (Thermo Fisher Scientific). Data-dependent fragmentation used collision-induced dissociation. RAW files were searched using MaxQuant under standard settings using the UniProt *Drosophila melanogaster* database, allowing for two missed trypsin cleavage sites, variable modifications for N-terminal acetylation, and methionine oxidation. Candidate peptides and protein identifications were filtered on the basis of a 1% false discovery rate.

### Secretome QC, differential abundance estimation, and functional enrichment

Raw intensities from the MaxQuant spectral database search engine were imported into R for downstream analysis. QC metrics such as number of unique peptides identified, percent of contaminants and reverse decoys detected, and total sum of intensities at the replicate level and condition level were computed and visualized using artMS v1.12.0. Spurious hits such as reverse decoys and potential contaminants were removed. R package DEP v1.16.0 was used to remove proteins not identified in both replicates simultaneously and variance stabilizing transformation was applied to normalize the intensities. Missing values were imputed using the k-nearest neighbors imputation method, followed by differential abundance estimation and multiple hypothesis testing correction in limma, all of which were implemented in DEP.

### Single cell RNA-seq data integration

#### Integration of Rust et. al. 2020 and Jevitt et. al. 2020

Previously published single cell RNA-sequencing datasets of the *Drosophila melanogaster* ovary from Rust et. al. and Jevitt et. al. aligned using CellRanger were obtained from the Gene Expression Omnibus database (GEO: GSE136162 and GSE146040 respectively). Seurat v4.1.1 was used to perform quality control, pre-processing, data integration, and differential expression analysis. Low quality cells were removed from individual datasets as described in the original publications prior to integration. The datasets were then individually normalized using sctransform implemented in Seurat using the glmGamPoi estimator for features that are present in at least 1 cell. Seurat RPCA (Reciprocal PCA) was used to integrate the two normalized datasets using an augmented feature list containing 3000 top ranking features from *SelectIntegrationFeatures* (Seurat) and a list of known FC and SFC marker genes. 100 neighbors (*k*.*anchors*) were used to achieve sufficient strength of integration in the *FindIntegrationAnchors* (Seurat) step [31]. Principal components were estimated for the integrated dataset and the top 30 dimensions were used to obtain the UMAP embeddings. New clusters were identified using the Louvain algorithm implemented in Seurat with the resolution set to 5. Cell identity labels from the original publications were retained in the integrated dataset to verify that clusters in the integrated dataset contain cells from analogous tissues in the original datasets.

#### Single Cell RNA-seq SFC and mid/late FC module score estimation, reclustering, and differential expression analysis

Seurat *AddModuleScore* was used to identify mid/late FC and SFC populations equivalent to those in the translatome and secretome datasets. To identify SFCs, previously published SFC-enriched genes *cv-2*, *dpp*, *eya*, *Past1*, *peb*, *drpr*, *puc*, *trol*, *kay*, *Vha16-1*, *Vha100-2* were used to compute an “SFCScore”. Similarly, to identify mid/late FCs, we scored cells using canonical markers such as *Yp1*, *Sox14*, and *br*. Cells with SFCScore >1 were designated as SFCs and cells with mid/late FC Score > 2 were designated as mid/late FCs. The integrated dataset was then subset to mid/late FC and SFC cells and reclustered. Differentially expressed genes between mid/late FC and SFC cells were identified using Seurat functions *PrepSCTFindMarkers* and *FindMarkers*, using the DESeq2 test with logfc.threshold parameter set to 0.25.

### DAPI and antibody staining

Ovaries were harvested in fresh 1X PBS and fixed at room temperature for 20 minutes in a solution of 4% paraformaldehyde (less than a week old), and 1X PBS, followed by two successive rinses in 1X PBT (1X PBS with 0.1% Triton X-100). The tissues were then washed 3 times in 1X PBT, each wash lasting 20 minutes. Following a rinse with 1X PBS, the tissues were incubated overnight at 4°C in 2 drops of Vectashield + 4’,6- diamidino-2-phenylindole dihydrochloride (DAPI) (Vector Laboratories) prior to mounting on slides.

Antibody staining was performed on ovaries following fixation and washing in PBT, as described above. Tissues were blocked in PBTG (PBT containing 1.5% normal goat serum) for 1 hour and then incubated in primary antibody diluted in PBTG, overnight at 4°C with agitation. The tissues were then washed in 4 times in PBT over 2 hours. The secondary antibody goat anti-mouse-Cy3 (Jackson Labs) was diluted in PBTG (1:200) and incubated with tissues for 2 hours at room temperature. The tissues were then washed 4 times in PBT and mounted in DAPI (Vector Labs). Primary antibodies were anti-V5 (Invitrogen 46–0705) used at 1:300 and anti-Discs large (Developmental Studies Hybridoma Bank 43F) used at 1:500. For detection of biotinylation, tissues were treated with biotin-phenol and H_2_O_2_ as described above before fixation and incubated with Streptavidin, Alexa Fluor 488 (Invitrogen).

### Imaging, quantification of mutant phenotypes, and statistics

DAPI stained tissues were imaged on an Olympus BX60 upright fluorescence microscope, Olympus FV10i confocal microscope or Nikon C2si confocal microscope. Image stacks were processed in FIJI and Adobe Illustrator. Figures, including drawings, were prepared in Adobe Illustrator. Stage 14 egg chambers were identified by the presence of a pair of fully formed dorsal appendages. The effect of disruption of phagoptosis of NCs resulting from RNAi perturbation of specific genes was quantified by the persistence of NC nuclei in stage 14 egg chambers. Persisting NC nuclei (PNCN) were visually identified and scored by binning the number of PNCN in each stage 14 egg chamber into 6 classes of phenotype severity, starting from egg chambers with 0 PNCN, and subsequently 1–3, 4–6, 7–9, 10–12, 13–15 PNCN. Egg chambers undergoing midstage death were identified by NC nuclear condensation and fragmentation as well as FC membrane enlargement (utilizing α-DLG staining). Midstage dying egg chambers were visually identified and quantified along with the number of germaria present per slide. The percentage of midstage death per ovariole was calculated by dividing the number of midstage dying eggs by the number of germaria and multiplied by 100. One-way ANOVA was used to calculate the statistical difference between both starved samples to the starved control and fed samples to the fed control. Those that had a p-value of < 0.05 were considered statistically significant.

## Supporting information

S1 Fig Analysis of secretome lysate and mass-spectrometry pre-processing. (A) (Top) Western blot analysis of lysate (30 μg per lane) from ovaries of indicated genotypes (GR1 –*GR1-GAL4*, HRP-KDEL–*UAS-HRP-KDEL-V5*, GR1>HRP-KDEL–*GR1-GAL4; UAS- HRP-KDEL-V5*, and PG150>HRP-KDEL–*PG150-GAL4; UAS-HRP-KDEL-V5*) incubated with or without substrate (biotin-phenol) and H_2_O_2_. *GR1>HRP-KDEL* and *PG150>HRP-KDEL* sample with both substrate and H_2_O_2_ have many biotinylated proteins as detected by streptavidin-HRP. (Bottom) α-V5 staining confirms HRP-KDEL expression in ovary tissue. (B) Western blot probed with streptavidin-HRP. Biotinylated proteins before (I- input, 30 μg) and after (B-beads, all of eluted protein) streptavidin enrichment. Genotypes as in G. (C) Scatterplot of peptide log_2_ intensity values of one replicate against another in each condition. (D) Distribution of log_2_ peptide intensity values in each replicate for all conditions.
(TIF)

S2 Fig Integration of scRNA-seq and comparison to the translatome. (A-D) Expression levels of canonical AFC and SFC markers. (E) Dot plot showing average expression and the percent of cells expressing each canonical marker in SFCs and AFCs. (F) Scatterplot of Log_2_ Fold Change values of differentially translated genes in the translatome (x-axis) and Log_2_ Fold Change (L2FC) values of differentially expressed genes in the integrated single cell RNA-seq AFC-SFC subset (y-axis). Positive values on both axes indicate upregulation in SFCs, when compared to the AFC baseline (denominator, negative values on both axes). The points are colored by whether the L2FC differential is significant at the adjusted p-value threshold of 0.05 in both, either, or neither comparison. (G) Summary of total number of genes in each category grouped by the direction of L2FC differential in scRNA-seq and translatome datasets. (H) Distribution of absolute value of differences in L2FC in translatome and scRNA-seq.
(TIF)

S3 Fig *in vivo* RNAi screening under protein-starved condition. (A-F) Egg chambers stained with DAPI (cyan) from the indicated genotypes under protein starvation. (A) *GR1>LexA RNAi* starved control shows sporadically degenerating egg chambers. (B-F) RNAi knockdowns of genes enriched in starved AFCs. (G) Quantitative analysis of midstage degenerating egg chambers with one-way ANOVA. (* p-value < 0.03, ** p-value < 0.009, ns p-value > 0.1) Graph displays mean + SD with n > 24 females per genotype and condition.
(TIF)

S1 Table GO-terms enriched in phagoptosis (Fed SFCs/Fed AFCs) and starvation-death (starved AFCs/Fed AFCs) identified in the translatome. Summary of the number of genes congruently (up or down) and uniquely regulated between SFCs and starved AFCs and the GO terms enriched in each category, along with the adjusted p-value.
(XLSM)

S2 Table Differentially regulated candidates (Log fold change and FDR adjusted p-values) in phagoptosis (Fed SFCs/Fed AFCs) across the translatome, secretome, and the integrated scRNA-seq datasets. Log fold change and FDR adjusted p-values obtained from DESeq2 (translatome, scRNA-seq) or DEP (secretome) are summarized for the SFC vs. fed AFC comparison.
(XLSM)

S3 Table Differentially regulated candidates in starvation-death (starved AFCs/Fed AFCs) across the translatome and secretome. Log fold change and FDR adjusted p-values obtained from DESeq2 (translatome) or DEP (secretome) are summarized for the starved AFC vs. fed AFC comparison.
(XLSM)

S4 Table Consolidated summary of candidates differentially expressed in the translatome in both phagoptosis (Fed SFCs/Fed AFCs) and starvation-death (Starved AFCs/Fed AFCs). Log fold change and FDR adjusted p-values obtained from DESeq2 are summarized for each gene in both SFC vs fed AFC comparison and starved AFC vs. fed AFC comparison. Genes are categorized as congruently up or down-regulated or as uniquely regulated in phagoptosis or starvation based on the fold change and FDR adjusted p-values across both comparisons.
(XLSM)

S5 Table Quantification of PNCN and mid-stage death in in vivo screening candidates. Raw data for Figs 5C, Fig 6C, and 7H showing number of stage 14 egg chambers categorized by the number of PNCN in genotypes listed in Table 1 are summarized in sheet 1 and raw data for Figs 6K and S3G Fig showing % mid-stage death per ovariole are summarized in sheet 2.
(XLSX)
