# Supplementary figures and images for: Multi-modal comparison of molecular programs driving nurse cell death and clearance in Drosophila melanogaster oogenesis
Source: PLoS Genet. 2025 Jan 3;21(1):e1011220. doi: 10.1371/journal.pgen.1011220 (PMC11734916; doi:10.1371/journal.pgen.1011220)

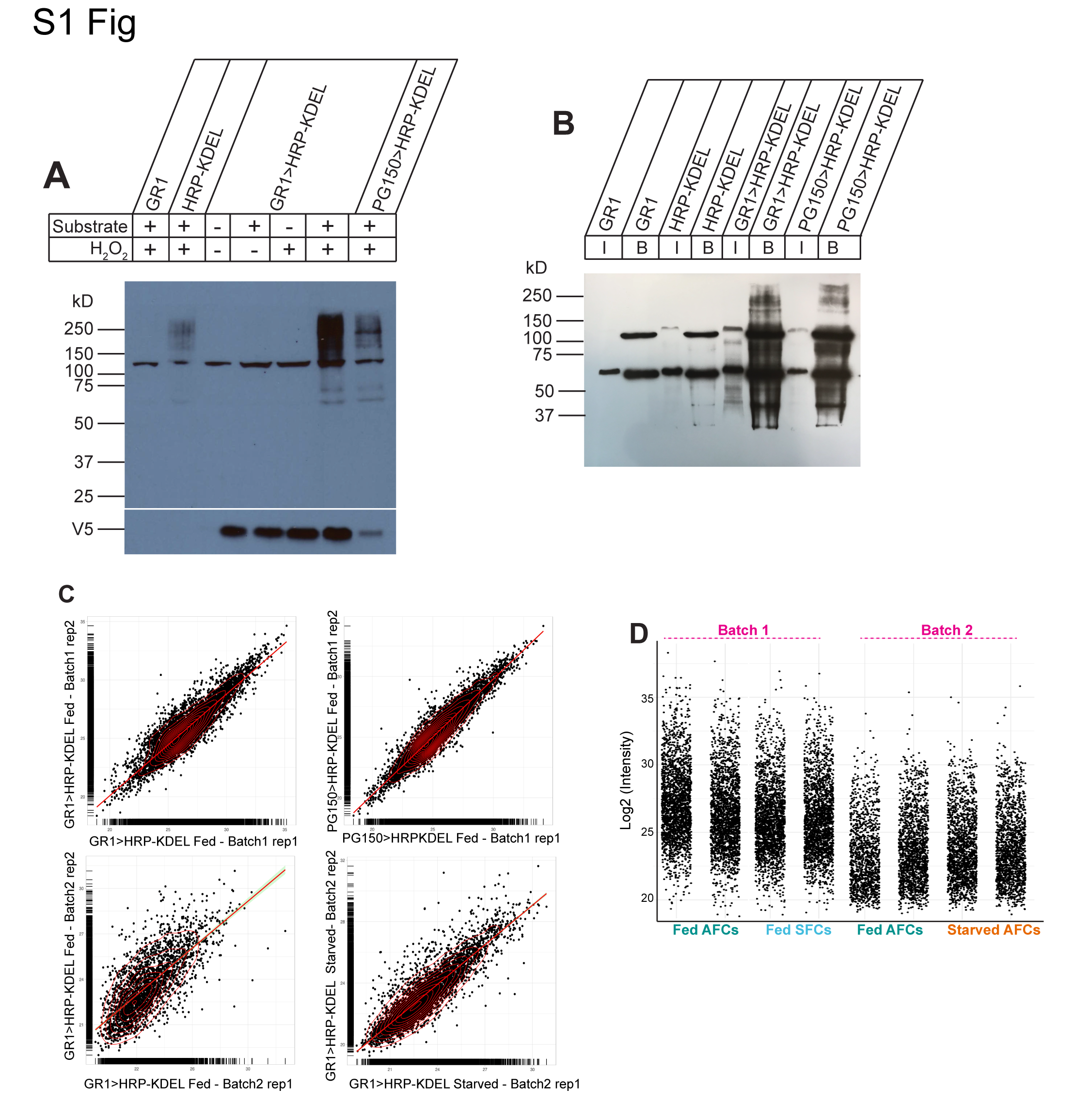

Supplement: S1 Fig — (A) (Top) Western blot analysis of lysate (30 μg per lane) from ovaries of indicated genotypes (GR1 –GR1-GAL4, HRP-KDEL–UAS-HRP-KDEL-V5, GR1>HRP-KDEL–GR1-GAL4; UAS- HRP-KDEL-V5, and PG150>HRP-KDEL–PG150-GAL4; UAS-HRP-KDEL-V5) incubated with or without substrate (biotin-phenol) and H2O2. GR1>HRP-KDEL and PG150>HRP-KDEL sample with both substrate and H2O2 have many biotinylated proteins as detected by streptavidin-HRP. (Bottom) α-V5 staining confirms HRP-KDEL expression in ovary tissue. (B) Western blot probed with streptavidin-HRP. Biotinylated proteins before (I- input, 30 μg) and after (B-beads, all of eluted protein) streptavidin enrichment. Genotypes as in G. (C) Scatterplot of peptide log2 intensity values of one replicate against another in each condition. (D) Distribution of log2 peptide intensity values in each replicate for all conditions. (TIF) [file pgen.1011220.s001.tif]

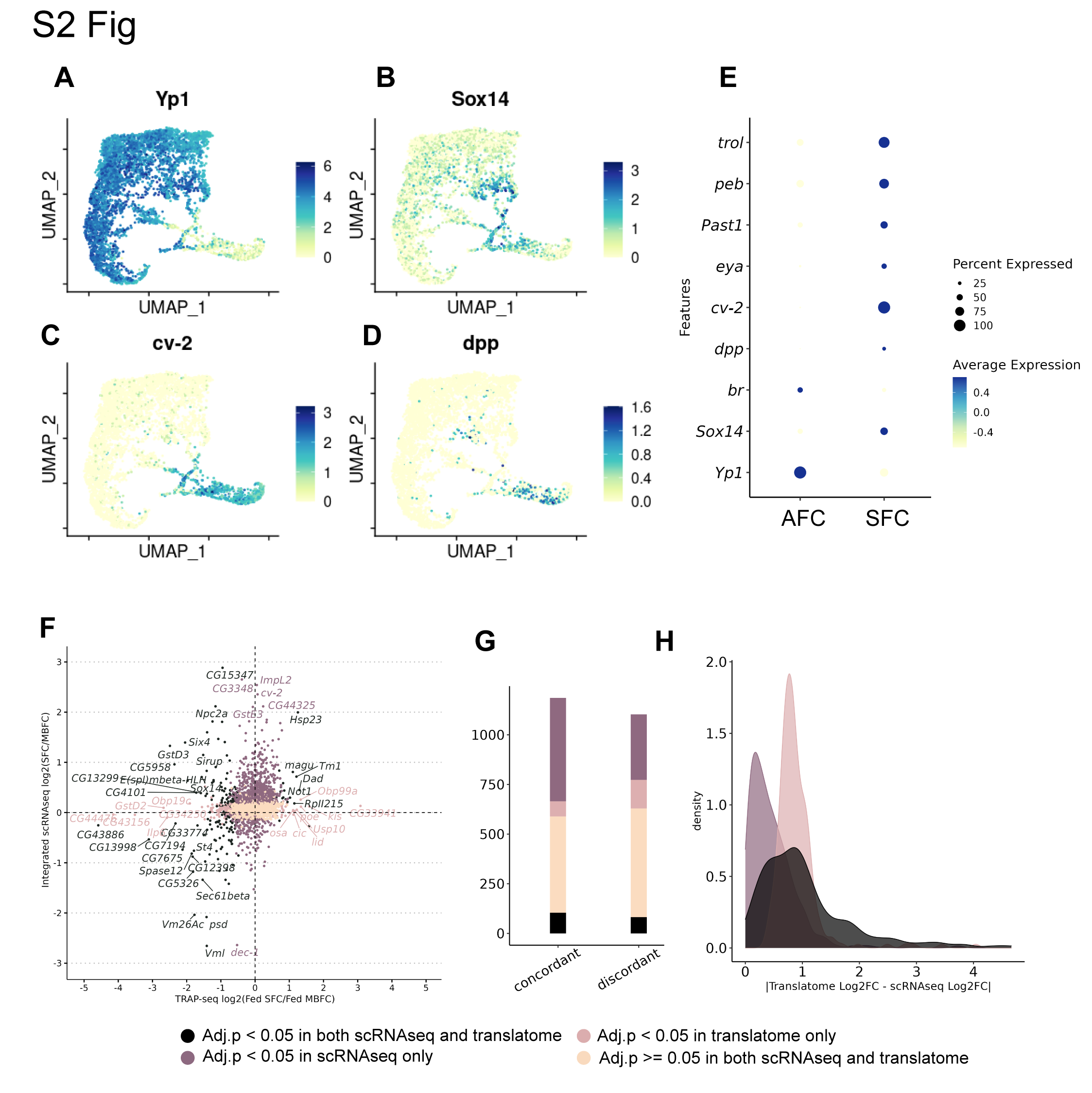

Supplement: S2 Fig — (A-D) Expression levels of canonical AFC and SFC markers. (E) Dot plot showing average expression and the percent of cells expressing each canonical marker in SFCs and AFCs. (F) Scatterplot of Log2 Fold Change values of differentially translated genes in the translatome (x-axis) and Log2 Fold Change (L2FC) values of differentially expressed genes in the integrated single cell RNA-seq AFC-SFC subset (y-axis). Positive values on both axes indicate upregulation in SFCs, when compared to the AFC baseline (denominator, negative values on both axes). The points are colored by whether the L2FC differential is significant at the adjusted p-value threshold of 0.05 in both, either, or neither comparison. (G) Summary of total number of genes in each category grouped by the direction of L2FC differential in scRNA-seq and translatome datasets. (H) Distribution of absolute value of differences in L2FC in translatome and scRNA-seq. (TIF) [file pgen.1011220.s002.tif]

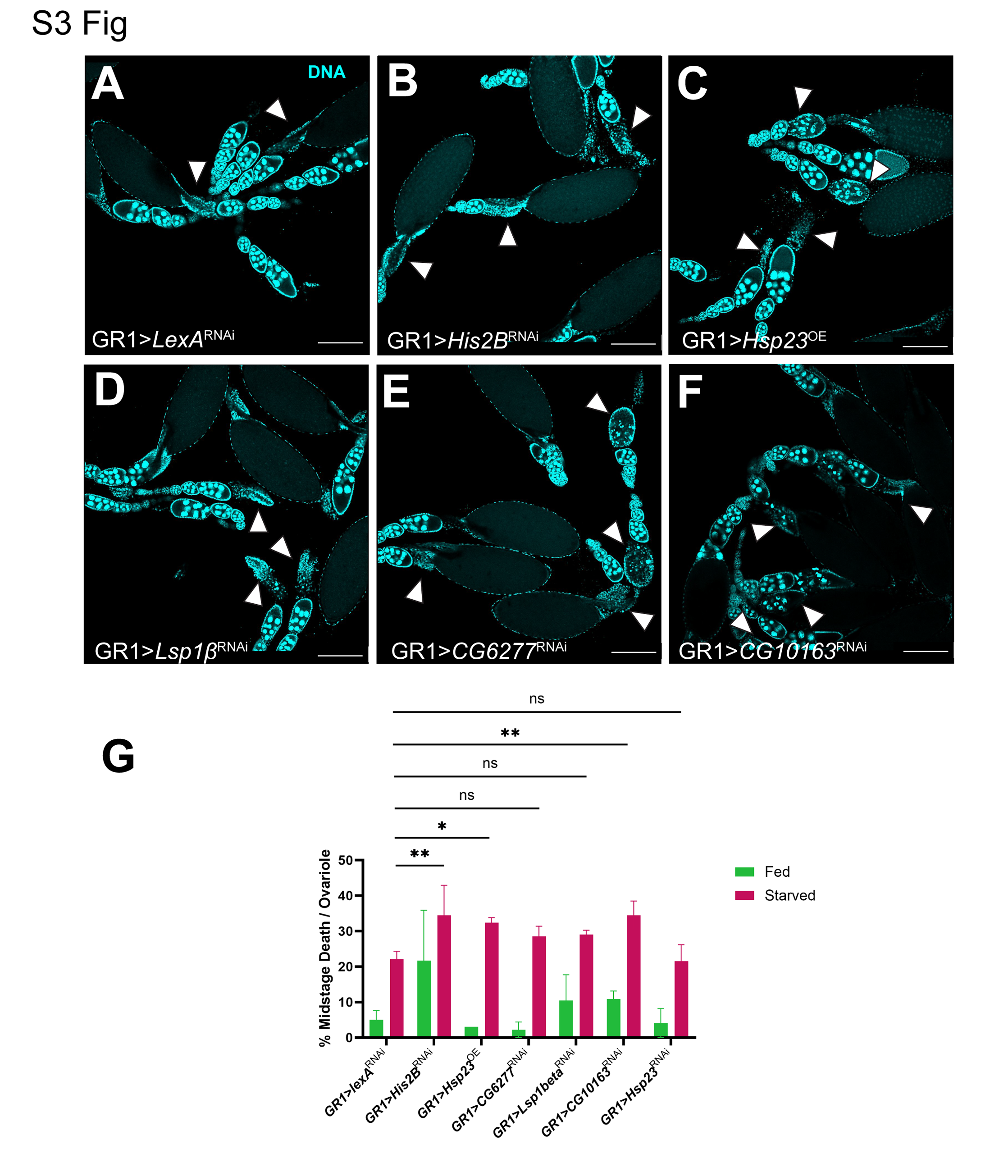

Supplement: S3 Fig — (A-F) Egg chambers stained with DAPI (cyan) from the indicated genotypes under protein starvation. (A) GR1>LexA RNAi starved control shows sporadically degenerating egg chambers. (B-F) RNAi knockdowns of genes enriched in starved AFCs. (G) Quantitative analysis of midstage degenerating egg chambers with one-way ANOVA. (* p-value < 0.03, ** p-value < 0.009, ns p-value > 0.1) Graph displays mean + SD with n > 24 females per genotype and condition. (TIF) [file pgen.1011220.s003.tif]
